# A Modified Artificial Protozoa Optimizer for Robust Parameter Identification in Nonlinear Dynamic Systems

**DOI:** 10.3390/biomimetics11010065

**Published:** 2026-01-12

**Authors:** Davut Izci, Serdar Ekinci, Gökhan Yüksek, Mostafa Rashdan, Burcu Bektaş Güneş, Muhammet İsmail Güngör, Mohammad Salman

**Affiliations:** 1 Department of Electrical and Electronic Engineering, Bursa Uludag University, Bursa 16059, Turkey; muhammetismailgungor@uludag.edu.tr; 2 Applied Science Research Center, Applied Science Private University, Amman 11931, Jordan; 3 Department of Computer Engineering, Bitlis Eren University, Bitlis 13100, Turkey; sekinci@beu.edu.tr; 4 Department of Electrical and Electronics Engineering, Batman University, Batman 72100, Turkey; gokhan.yuksek@batman.edu.tr; 5 College of Engineering and Technology, American University of the Middle East, Egaila 54200, Kuwait; mohammad.salman@aum.edu.kw; 6 Department of Computer Engineering, Istanbul Gedik University, İstanbul 34876, Turkey; burcu.gunes@gedik.edu.tr

**Keywords:** artificial protozoa optimizer, Nelder–Mead simplex method, random learning mechanism, nonlinear systems, parameter identification

## Abstract

Accurate parameter identification in nonlinear and chaotic dynamic systems requires optimization algorithms that can reliably balance global exploration and local refinement in complex, multimodal search landscapes. To address this challenge, a modified artificial protozoa optimizer (mAPO) is developed in this study by embedding two complementary mechanisms into the original artificial protozoa optimizer: a probabilistic random learning strategy to enhance population diversity and global search capability, and a Nelder–Mead simplex-based local refinement stage to improve exploitation and fine-scale solution adjustment. The general optimization performance and scalability of the proposed framework are first evaluated using the CEC2017 benchmark suite. Statistical analyses conducted over shifted and rotated, hybrid, and composition functions demonstrate that mAPO achieves improved mean performance and reduced variability compared with the original APO, indicating enhanced robustness in high-dimensional and complex optimization problems. The effectiveness of mAPO is then examined in nonlinear system identification applications involving chaotic dynamics. Offline and online parameter identification experiments are performed on the Rössler chaotic system and a permanent magnet synchronous motor, including scenarios with abrupt parameter variations. Comparative simulations against APO and several state-of-the-art optimizers show that mAPO consistently yields smaller objective function values, more accurate parameter estimates, and superior statistical stability. In the PMSM case, exact parameter reconstruction with zero error is achieved across all independent runs, while rapid and smooth convergence is observed under both static and time-varying conditions.

## 1. Introduction

Accurate parameter identification plays a central role in the analysis, modeling, and control of nonlinear dynamic systems. When the underlying dynamics are chaotic or strongly nonlinear, even small parameter deviations can produce large discrepancies in the observed trajectories, making reliable estimation essential for prediction, stabilization, and controller design. In many practical applications (ranging from electromechanical drives to biological systems and secure communication schemes), the governing parameters are difficult to measure directly and may vary over time due to environmental conditions, component aging, or abrupt disturbances. For these reasons, parameter identification has become a fundamental problem in nonlinear system theory and continues to attract considerable attention across the optimization and control communities [1,2,3].

Traditional mathematical tools for parameter estimation often rely on gradient-based procedures or linearization around equilibrium points. Although such approaches may perform adequately for mildly nonlinear models, they tend to struggle when confronted with nonconvex cost landscapes, multimodal error surfaces, or chaotic attractors [4]. Chaotic systems, in particular, present unique challenges: their sensitivity to initial conditions and inherent structural complexity produce rugged optimization landscapes with numerous deceptive minima, causing deterministic optimizers to converge prematurely or diverge entirely [5]. As a result, metaheuristic algorithms (characterized by their global search capability, population-based structure, and independence from gradient information [6]) have become increasingly popular alternatives for solving such identification problems.

### 1.1. Existing Studies on Nonlinear and Chaotic Parameter Identification

A wide range of metaheuristic strategies has been proposed to address nonlinear and chaotic identification problems, including evolutionary algorithms [7], swarm intelligence methods [8], physics-inspired optimizers [9], and hybridized techniques [4] designed to balance exploration and exploitation more effectively. Examples include the flower pollination algorithm [10], improved fruit fly optimizer [11], quantum-enhanced particle swarm optimization [12], and hybrid differential evolution–artificial bee colony algorithm [13]. These methods have achieved notable success in several identification tasks; however, they often continue to exhibit sensitivity to initial conditions, premature convergence, limited local search capability, and instability in highly nonlinear regions of the parameter space.

A considerable amount of research has examined nonlinear and chaotic systems, with the Rössler attractor frequently adopted as a benchmark for evaluating modeling, prediction, and identification methods due to its structurally simple formulation and rich dynamical behavior [14]. Several learning- and intelligence-based approaches have been tested on Rössler data to assess their capability to reconstruct or predict chaotic dynamics. For instance, an interval type-2 neo-fuzzy neural network was shown in [15] to model complex dynamics effectively, where both the Mackey–Glass and Rössler systems were used as test cases. The study demonstrated that although fuzzy neural networks can successfully learn short-term chaotic behavior, their performance is strongly influenced by training stability and the tuning of adaptive parameters, highlighting the difficulty of maintaining accuracy in the presence of chaotic sensitivity.

Similarly, a data-driven identification methodology was explored in [16], where recursive estimation techniques were applied to nonlinear chaotic systems, including the Rössler attractor. The work showed that estimation performance deteriorates significantly when the system parameters drift or when noise levels increase, reinforcing the importance of robust optimization procedures. In another line of research, sparse regression and structural discovery methods have been investigated for chaotic models. The approach described in [17] attempted to recover the governing equations of hyperchaotic Rössler dynamics through stepwise model refinement, but its accuracy declined under noisy conditions or abrupt parameter changes.

Fuzzy-logic-based modeling has also been used to approximate chaotic trajectories. In Ref. [18], a type-2 neo-fuzzy neural network was trained on Rössler time series to evaluate its prediction capability, showing improved modeling flexibility but also revealing dependence on suitable learning strategies. Collectively, these studies indicate that although a wide range of intelligent and data-driven methods have been applied to Rössler-type systems, many existing techniques suffer from premature convergence, insufficient robustness, and degraded accuracy when confronted with chaotic error surfaces. These persistent limitations underscore the need for optimization frameworks that can maintain global search ability while offering precise local refinement for parameter identification.

Parallel challenges arise in the parameter identification of permanent magnet synchronous motors (PMSMs) [19], which exhibit nonlinear electromechanical interactions and are sensitive to temperature, magnetic saturation, and inverter nonlinearities. Accurate PMSM parameter estimation is essential for ensuring stable torque production, efficient field-oriented control, and reliable operation under dynamic conditions. Several nonlinear modeling frameworks have been applied to PMSM systems. The comprehensive survey in [20] highlighted the capability of multi-dimensional Taylor networks (MTNs) to approximate motor dynamics and improve prediction and control performance. Since MTN-based identification accuracy depends critically on the quality of the optimization routine, this further emphasizes the central role of robust parameter-estimation techniques. Advanced control structures have also been explored. In Ref. [21], predictive control architectures tailored to PMSMs were reviewed, and it was shown that even sophisticated control schemes remain highly sensitive to parameter mismatch. The work stressed that inaccurate motor parameters can significantly impair dynamic response, thereby requiring reliable identification methods capable of tracking parameter variations in real time.

To address these issues, numerous optimization-driven identification strategies have been proposed. For example, the asynchronous niche particle swarm optimization (AN-PSO) method introduced in [22] incorporated adaptive step-size and population-diversity mechanisms, achieving accurate online estimation even under inverter nonlinearities. The study also highlighted the weaknesses of traditional PSO (particularly its tendency to stagnate in local minima) thereby motivating hybrid and improved variants. Furthermore, Ref. [23] presented a noninvasive parameter estimation method for PMSMs without signal injection, designed to operate under limited operating-condition variability. Although effective, this method still faced challenges when confronted with parameter variations and imperfect inverter-nonlinearity compensation. The authors noted that optimization-based techniques could further enhance estimation accuracy, particularly in systems exhibiting pronounced nonlinear behavior.

Taken together, the literature on both chaotic models such as the Rössler system and nonlinear electromechanical systems such as PMSMs points to a shared methodological gap. Despite notable progress, many metaheuristic algorithms still struggle to maintain a reliable balance between global exploration and local refinement, particularly when dealing with multimodal or highly sensitive dynamical landscapes. The performance of existing identification methods remains heavily dependent on the underlying optimization engine, and most continue to experience premature convergence, noise sensitivity, or reduced adaptability when parameters vary abruptly.

### 1.2. Empirical Contribution and Innovation of the Present Study

Motivated by the limitations identified in the existing literature, the present study proposes a modified version of the artificial protozoa optimizer (APO) [24], designed specifically to improve robustness, accuracy, and convergence reliability in nonlinear optimization and parameter identification problems. The novelty of the proposed method lies in its hybrid search architecture, which is achieved by embedding two complementary mechanisms into the original APO framework. First, a probabilistic random learning strategy [25] is incorporated to enrich global exploration by injecting diversity informed by both the global best solution and population-based difference information. Second, a Nelder–Mead simplex refinement stage [26] is introduced to enhance local exploitation by enabling efficient fine-scale adjustment around promising candidate solutions. When combined with the biological behaviors inherent in the artificial protozoa optimizer, these mechanisms yield a more adaptive search process capable of escaping deceptive local minima while still converging toward high-precision solutions. This hybridization is particularly valuable for chaotic and nonlinear problems, where effective optimization requires both broad exploration and meticulous refinement.

To establish the empirical effectiveness and generalization capability of the proposed modified artificial protozoa optimizer (mAPO), a two-stage evaluation strategy is adopted. First, the general optimization performance of mAPO is assessed using the CEC2017 benchmark function suite [27], which is widely recognized for evaluating optimization algorithms in high-dimensional, multimodal, hybrid, and composition-based search landscapes. This benchmark analysis is conducted to examine scalability, robustness, and statistical stability under challenging optimization conditions that are independent of any specific system model. The results obtained from the CEC2017 benchmarks demonstrate that the proposed modifications lead to improved mean performance and reduced variability when compared with the original APO, indicating that the benefits of the hybrid search structure extend beyond application-specific identification tasks.

Following this general validation, the proposed framework is applied to nonlinear parameter identification problems involving chaotic dynamics. Two benchmark systems exhibiting nonlinear and chaotic behavior are examined: the Rössler chaotic system [28] and a permanent magnet synchronous motor [29], which is known to display chaotic oscillatory patterns under certain parameter conditions. Both offline parameter identification and online tracking scenarios are investigated to evaluate estimation accuracy, convergence speed, statistical consistency, and adaptability to abrupt parameter variations. The results show that mAPO consistently outperforms existing metaheuristic algorithms, achieving extremely small objective function values, reproducing the true parameter sets with numerical precision, and maintaining exceptional stability across independent runs. The observed convergence profiles and adaptive behavior further confirm the effectiveness of the integrated random learning and simplex refinement strategies.

Taken together, these empirical investigations demonstrate that the proposed mAPO framework provides a robust and accurate optimization strategy that is effective in both high-dimensional benchmark optimization problems and nonlinear chaotic parameter identification tasks. The results underscore the practical value of unifying structured local search with biologically inspired metaheuristic exploration and position mAPO as a versatile and reliable optimization tool for complex nonlinear systems.

### 1.3. Paper Organization

The remainder of this paper is organized to present the proposed methodology and its evaluation in a clear and systematic manner. Section 2 formulates the nonlinear parameter identification problem and introduces the two benchmark systems considered in this study, namely the chaotic Rössler system and the permanent magnet synchronous motor, which serve as representative nonlinear and chaotic dynamic models. Section 3 provides an overview of the original artificial protozoa optimizer, describing its fundamental biological behaviors and the associated mechanisms governing foraging, dormancy, reproduction, and behavior switching. Section 4 then introduces the proposed modified artificial protozoa optimizer, where the probabilistic random learning strategy and the integration of the Nelder–Mead simplex search are detailed, followed by a description of the overall working mechanism of the enhanced algorithm. Section 5 presents the simulation results and performance evaluations. First, the general optimization capability of the proposed mAPO is assessed using the CEC2017 benchmark function suite. Subsequently, the parameter identification results for the chaotic Rössler system are reported, including statistical analyses, convergence behavior, parameter evolution, accuracy assessment, comparisons with reported methods, and online identification performance. The same evaluation framework is then applied to the permanent magnet synchronous motor system, where offline and online identification results are analyzed and compared in detail. Section 5.4 discusses the obtained results by synthesizing the findings from the benchmark optimization tests and the nonlinear system identification studies, highlighting the robustness, accuracy, and adaptability of the proposed framework, as well as its limitations. Finally, Section 6 concludes the paper by summarizing the main outcomes and emphasizing the novelty and practical significance of the proposed hybrid optimization approach.

## 2. Parameter Identification of Nonlinear System

### 2.1. Problem Statement

The identification of unknown parameters in nonlinear dynamic systems may be formulated as an optimization problem in which the goal is to minimize the mismatch between the measured system response and the trajectory generated by its mathematical model. Let $X_{k}\in R^{n}$ denote the true state vector of the system at sampling instant k, and ${\hat{X}}_{k}={\hat{X}}_{k}(\theta )$ represent the simulated state vector produced by integrating the model with a candidate parameter vector θ. For each set of parameters, the deviation between the measured and simulated trajectories is quantified using the mean squared error (*MSE*) objective function defined in Equation (1):

(1) $$J=MSE=\frac{1}{M}\sum_{k=1}^{M}{\left\|X_{k}-{\hat{X}}_{k}\right\|}^{2}$$

In this formulation, M denotes the number of sampled data points, $X_{k}$ corresponds to the reference (measured) system states, ${\hat{X}}_{k}$ represents the model-generated states under the current parameter estimates, and J evaluates the cumulative modeling error over the sampling horizon.

**Figure 1:**
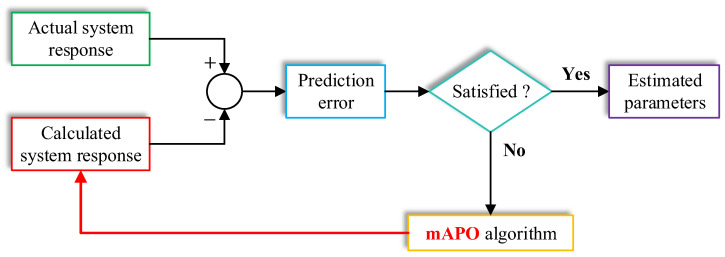
Parameter estimation procedure using mAPO for nonlinear system.

The task of parameter identification is therefore reduced to determining the parameter vector θ that minimizes J. A lower objective value indicates a closer resemblance between the model and the actual nonlinear system, whereas larger values imply that the candidate parameters fail to reproduce the underlying dynamics with sufficient accuracy. The overall estimation process is illustrated schematically in Figure 1, which outlines the procedure followed by the modified artificial protozoa optimizer. As shown, the measured state sequence $X_{k}$ is first supplied to the estimation framework. The optimizer then generates an initial population of candidate parameter vectors, each of which is evaluated by simulating the nonlinear system to obtain ${\hat{X}}_{k}$. The corresponding objective value is computed through Equation (1), and the optimizer iteratively updates the candidate parameters to reduce this error. Throughout the process, exploration enables the search to cover a broad region of the parameter space, while exploitation refines the solutions toward the parameter sets that yield improved model fidelity. This iterative cycle continues until the convergence criteria are met, after which the candidate producing the smallest objective function value is accepted as the final estimate. By structuring the identification problem in this manner, the optimizer is guided directly by the system’s measurable behavior, ensuring that the estimated parameters accurately reconstruct the nonlinear dynamics of the underlying model.

### 2.2. Test System I: Chaotic Rössler System

The first test platform considered in this study is the classical chaotic Rössler system, which has long served as a benchmark model for evaluating parameter identification techniques due to its structurally simple formulation and rich nonlinear behavior [30,31]. The system, originally introduced by Rössler as a minimal representation of continuous-time chaos, contains only a single nonlinear term and exhibits the characteristic spiral-type attractor widely discussed in the literature [32]. The governing dynamics are expressed through the coupled differential equations given below:

(2) $$\frac{dx}{dt}=-y-z$$

(3) $$\frac{dy}{dt}=x+\sigma y$$

(4) $$\frac{dz}{dt}=\rho +z(x-\beta )$$

where x, y, and z denote the state variables of the system, and σ, ρ, and β are the unknown parameters to be estimated. These parameters directly influence the stretching and folding mechanisms that give rise to the system’s strange attractor, a feature well documented in foundational studies on continuous chaos.

To conduct parameter estimation experiments, the unknown coefficients were constrained within predefined admissible ranges of 0≤σ≤1, 0≤ρ≤1 and 0≤β≤10. The true parameter values used to generate the reference trajectory were selected as σ=0.2, ρ=0.2 and β=5.7 which correspond to a well-known chaotic regime of the Rössler dynamics. The associated initial conditions were chosen as x(0)=1, y(0)=1 and z(0)=1 ensuring that the system evolves toward the characteristic spiral attractor.

**Figure 2:**
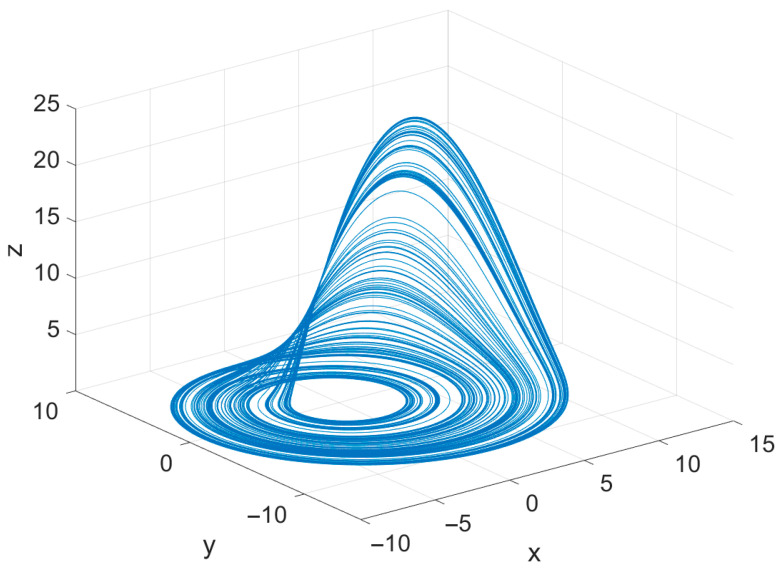
Three-dimensional phase portrait of the Rössler chaotic system generated from the mathematical model for σ=0.2, ρ=0.2, and β=5.7.

For numerical integration, a fourth-order Runge–Kutta (RK4) method was employed with a fixed step length of h=0.01. This discretization is consistent with common practice in the literature, as it provides a reliable balance between computational cost and integration accuracy for chaotic flows. A total of M=300 sampling points was extracted from the simulated trajectory and subsequently used for objective function evaluation during the optimization process. The resulting three-dimensional phase portrait of the system, generated using the actual parameter set, is presented in Figure 2. The figure illustrates the classical Rössler attractor reflected in a folded, disk-like geometry, where trajectories spiral outward before being redirected inward through the nonlinear interaction term. This structure highlights the sensitivity of the system to its governing parameters, thereby making it an appropriate test environment for assessing the robustness and precision of the proposed mAPO-based parameter estimation methodology. Overall, the Rössler system provides a controlled yet dynamically rich benchmark that enables rigorous evaluation of the optimizer’s ability to recover the underlying parameters from observed state trajectories.

### 2.3. Test System II: Permanent Magnet Synchronous Motor (PMSM) System

The second test system employed in this study is the chaotic permanent magnet synchronous motor (PMSM). PMSMs are widely used in industrial applications due to their high efficiency and favorable torque–speed characteristics; however, it is well established that the motor may exhibit chaotic oscillations when its internal parameters enter specific ranges [33]. Such behavior has been documented in several studies, where irregular current and speed trajectories emerge even under nominal conditions. This makes the PMSM an appropriate benchmark for evaluating parameter estimation algorithms, particularly when the goal is to recover unknown parameters from nonlinear and potentially chaotic dynamics [34].

In this work, the PMSM model is considered in its standard three-state form, consisting of the direct-axis current x, the quadrature-axis current y, and the rotor speed z. The system dynamics adopted in the manuscript are expressed as:

(5) $$\frac{dx}{dt}=-x+yz$$

(6) $$\frac{dy}{dt}=-y-xz+\sigma z$$

(7) $$\frac{dz}{dt}=z+\rho (y-z)$$

where x, y, and z represent the measurable state variables of the PMSM, σ and ρ denote the unknown system parameters to be identified. These equations correspond to the widely used reduced-order PMSM representation reported in the PMSM chaos literature, in which the nonlinear coupling terms are responsible for the onset of chaotic motion under certain parameter combinations. Because of this, the system provides a challenging yet informative test case for assessing the performance of the modified artificial protozoa optimizer (mAPO).

For the parameter identification study, the unknown coefficients were restricted to the admissible bounds of 10≤σ≤30 and 1≤ρ≤10 ensuring that the search process remains within meaningful physical limits while still allowing the optimizer to navigate a sufficiently large parameter space. The true parameter values used to generate the reference trajectory were selected as σ=20 and ρ=5.46 and the system was initialized with the state conditions x(0)=5, y(0)=1 and $z\left(0\right)=-1$. To reduce computational burden while maintaining numerical accuracy, the system was integrated using the fourth-order Runge–Kutta method, with the step length, specified as h=0.001. A set of M=300 sampling points was then extracted from the resulting trajectory for evaluation through the objective function.

**Figure 3:**
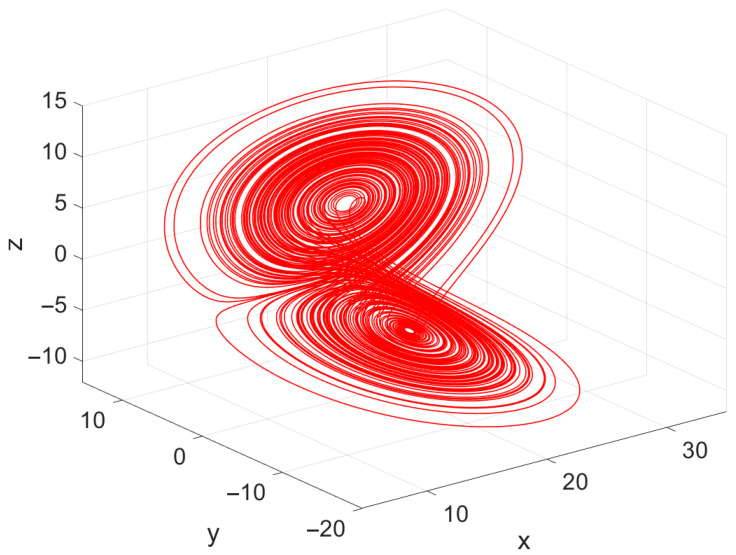
Three-dimensional phase portrait of the PMSM system generated from the mathematical model for σ=20 and ρ=5.46.

The corresponding three-dimensional phase portrait of the PMSM, generated using the true parameter set, is shown in Figure 3. The attractor displays the characteristic folded, non-periodic structure associated with chaotic PMSM dynamics. Such behavior highlights the system’s sensitivity to its internal parameters and underscores the importance of accurate parameter identification when stable and predictable motor operation is desired. Overall, the PMSM system provides a demanding test environment for the proposed mAPO, with its nonlinear couplings and potential for chaotic oscillations offering a rigorous benchmark for evaluating the algorithm’s identification accuracy and robustness.

## 3. Artificial Protozoa Optimizer

The artificial protozoa optimizer (APO) models the adaptive survival behaviors of unicellular protozoa (specifically Euglena) and formulates them into a set of mathematical operators suitable for continuous optimization [24]. The optimizer simulates three characteristic behaviors: foraging, dormancy, and reproduction, each contributing differently to the balance between exploration and exploitation. The population consists of $p_{s}$ protozoa, where each protozoan represents a solution vector in a dim-dimensional decision space.

### 3.1. Foraging Behavior

Protozoa obtain nutrients either autotrophically (light-driven) or heterotrophically (via organic matter). These two behaviors are modelled separately to capture different movement dynamics in the search space.

#### 3.1.1. Autotrophic Foraging (Exploration)

In this mode, a protozoan adjusts its position relative to a reference protozoan and its neighborhood. The autotrophic update equation is:

(8) $$X_{i}^{\;\mathrm{new}}=X_{i}+f\cdot \left(X_{j}-X_{i}+\frac{1}{n_{p}}\sum_{k=1}^{n_{p}}w_{a}\cdot (X_{k^{-}}-X_{k^{+}})\right)⊙M_{f}$$

where $X_{i}$ is current protozoan position, $X_{j}$ is a randomly selected protozoan, $X_{k^{-}}$, $X_{k^{+}}$ are paired neighbors with rank indices lower and higher than i, $M_{f}$ is mapping vector specifying which dimensions will be updated. f is foraging factor, defined as $\mathrm{rand}\cdot (1+cos\left(iter/{\mathrm{iter}}_{max}\right)\pi )$. The maximum number of neighbor pairs are given as $n_{p_{max}}=\left(\left(p_{s}-1\right)/2\right)$. The autotrophic weight factor is calculated as follows:

(9) $$w_{a}=e^{\left(-\left|\frac{f(X_{k^{-}})}{f(X_{k^{+}})+\varepsilon }\right|\right)}$$

with $\varepsilon =2.2204\times 10^{-16}$. The mapping vector $M_{f}$ determines which decision variables participate in the movement and is defined as $M_{f}\left[d_{i}\right]=1$ for $d_{i}\in \mathrm{randperm}(\dim,\;\left\lceil \dim\cdot \left(i/p_{s}\right)\right\rceil )$ and $M_{f}\left[d_{i}\right]=0$ for otherwise. These components allow autotrophic foraging to produce broad search steps and contribute primarily to exploration.

#### 3.1.2. Heterotrophic Foraging (Exploitation)

In darker conditions, protozoa move toward nearby nutrient-rich areas. The heterotrophic update rule is:

(10) $$X_{i}^{\;\mathrm{new}}=X_{i}+f\cdot \left(X_{\mathrm{near}}-X_{i}+\frac{1}{n_{p}}\sum_{k=1}^{n_{p}}w_{h}\cdot (X_{i-k}-X_{i+k})\right)⊙M_{f}$$

where the nearby target is computed as $X_{\mathrm{near}}=\left(1\pm \mathrm{Rand}\cdot (1-\left(iter/{\mathrm{iter}}_{max}\right))\right)⊙X_{i}$, $\mathrm{Rand}=[{\mathrm{rand}}_{1},{\mathrm{rand}}_{2},\ldots ,{\mathrm{rand}}_{\dim}]$, and the heterotrophic weight factor is defined as follows.

(11) $$w_{h}=e^{\left(-\left|\frac{f(X_{i-k})}{f(X_{i+k})+\varepsilon }\right|\right)}$$

This model narrows the search region over time and strengthens local refinement, giving heterotrophic foraging its exploitative nature.

### 3.2. Dormancy Mechanism (Exploration)

When conditions deteriorate, protozoa may enter a dormant state. In APO, dormancy is simulated by regenerating new individuals within the search bounds:

(12) $$X_{i}^{\;\mathrm{new}}=X_{min}+\mathrm{Rand}⊙(X_{max}-X_{min})$$

where $X_{min}=\left[lb_{1},lb_{2},\ldots ,lb_{\dim}\right],X_{max}=[ub_{1},ub_{2},\ldots ,ub_{\dim}]$. Dormancy injects fresh diversity and helps the population escape stagnation.

### 3.3. Reproduction Mechanism (Exploitation)

Protozoa reproduce through binary fission, which is simulated by perturbing the current position:

(13) $$X_{i}^{\;\mathrm{new}}=X_{i}\pm \mathrm{rand}\cdot \left(X_{min}+\mathrm{Rand}⊙(X_{max}-X_{min})\right)⊙M_{r}$$

where the reproduction mapping vector $M_{r}$ is defined as $M_{r}[d_{i}]=1$ for $d_{i}\in \mathrm{randperm}(\dim,\;\left\lceil \dim\cdot \mathrm{rand}\right\rceil )$ and $M_{r}\left[d_{i}\right]=0$ for otherwise. This operator introduces controlled directional modifications that intensify the search around promising regions.

### 3.4. Behavior-Switching Probabilities

The APO switches adaptively between foraging, dormancy, and reproduction using three probability functions:

(14) $$p_{f}=p_{f_{max}}\cdot \mathrm{rand}$$

(15) $$p_{ah}=\frac{1}{2}\left(1+cos\left(\frac{\mathrm{iter}}{{\mathrm{iter}}_{max}}\pi \right)\right)$$

(16) $$p_{dr}=\frac{1}{2}\left(1+cos\left(\left(1-\frac{i}{p_{s}}\right)\pi \right)\right)$$

where $p_{ah}$ switches between autotrophic and heterotrophic foraging, $p_{dr}$ switches between dormancy and reproduction, and $p_{f}$ determines the proportion of protozoa undergoing survival-related behaviors. These adaptive probabilities help transition the algorithm from exploration-dominant behavior in early iterations to exploitation-focused refinement later.

## 4. Modified Artificial Protozoa Optimizer

Although the APO offers a flexible balance between exploration and exploitation through its biological behavior model, its performance can deteriorate when the population approaches narrow, curved, or locally complex regions of the search space. In such cases, the original operators may fail to intensify the search effectively, resulting in slow convergence or entrapment in regions around local optima. To address these limitations, an enhanced version, referred to as the modified APO (mAPO), is developed by embedding two additional mechanisms within the APO framework: (1) a random learning strategy that strengthens adaptive exploration [35], and (2) a local refinement stage based on the Nelder–Mead simplex method [36], which reinforces exploitation once a promising region has been located.

### 4.1. Random Learning Strategy

**Figure 4:**
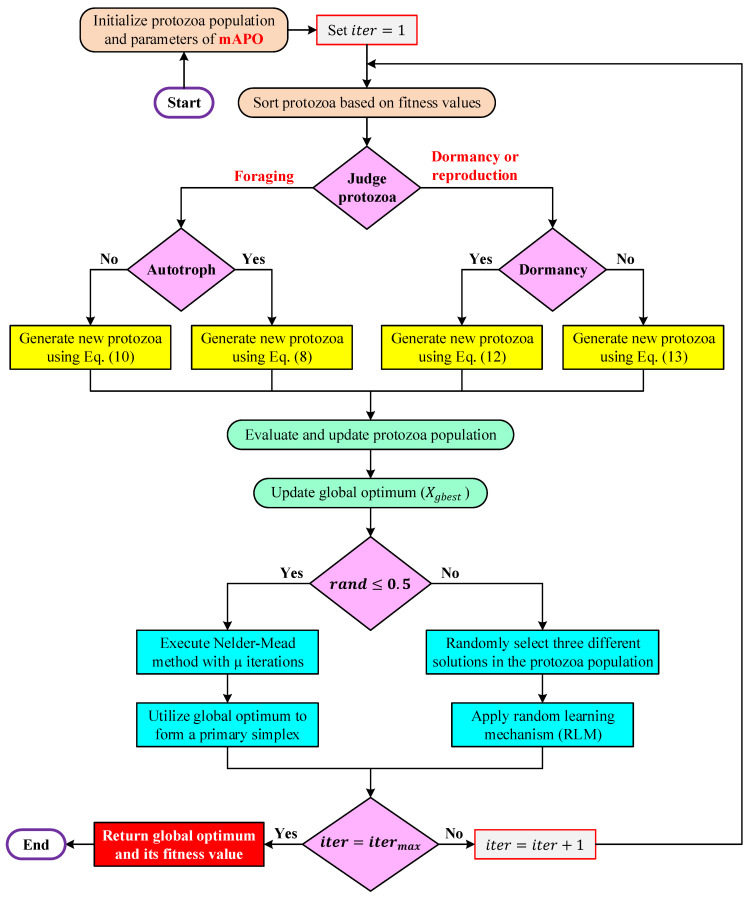
Flowchart of mAPO algorithm.

The first enhancement introduces a probabilistic learning mechanism that periodically perturbs selected protozoa based on information extracted from the best solutions discovered so far. When activated, the update acts as an additional exploratory jump, enabling the optimizer to diversify the population and prevent stagnation around suboptimal regions. For a protozoan $X_{i}$, the random learning update (RLS) [37] is defined as:

(17) $$X_{i}^{\mathrm{RLS}}=X_{i}+\eta \cdot (X_{\mathrm{best}}-X_{i})+\gamma \cdot \left(X_{j}-X_{k}\right)$$

where $X_{\mathrm{best}}$ is the best individual in the population, $X_{j}$ and $X_{k}$ are two distinct randomly selected protozoa, η and γ are random factors drawn from [0,1]. The first term encourages guided learning from the global best solution, while the second introduces lateral exploration derived from population diversity [38]. As a result, the optimizer becomes more resilient to early convergence and gains the ability to escape shallow local minima. The activation of RLS in Figure 4 is shown to take place after the behavioral operator (foraging, dormancy, or reproduction) is executed, ensuring that it serves as a corrective, diversity-enhancing step.

### 4.2. Integration of Nelder–Mead Simplex Search

The second modification embeds a local search operator based on the Nelder–Mead (NM) simplex method [39], a derivative-free algorithm well suited for fine-tuning candidate solutions within continuous spaces. In mAPO, NM is triggered selectively when the best individual exhibits stagnation over several iterations, indicating that additional exploitation is required. Given a protozoan $X_{\mathrm{best}}$ designated for refinement, a simplex comprising dim + 1 vertices is constructed in its vicinity. The NM method then performs reflection, expansion, contraction, or shrinkage operations according to the classical rules known as reflection (Equation (18)), expansion (Equation (19)), and contraction (Equation (20)):

(18) $$X_{r}=X_{c}+\alpha \;(X_{c}-X_{w})$$

(19) $$X_{e}=X_{c}+\beta \;(X_{r}-X_{c})$$

(20) $$X_{con}=X_{c}+\delta \;(X_{w}-X_{c})$$

where $X_{c}$ is the centroid of the simplex excluding the worst vertex $X_{w}$, α, β, and δ are the reflection, expansion, and contraction coefficients, respectively. By incorporating NM, mAPO is able to conduct precise local search around high-quality regions identified by APO’s global mechanisms. This interaction between biologically inspired movement and simplex-based refinement produces a synergistic effect: APO drives the population toward promising zones, and NM accelerates convergence once the region of interest is reached. In Figure 4, the NM search module is placed after the random learning block, confirming that local refinement is performed only after candidate solutions have undergone both biological and stochastic updates.

In accordance with the commonly adopted configuration reported in the NM optimization literature, the simplex coefficients were fixed as follows: reflection factor α=1, expansion factor β=2, and contraction factor δ=0.5. These values are widely used in derivative-free local search due to their favorable balance between convergence speed and numerical stability [38]. In the present study, the coefficients were kept constant across all simulations to ensure consistency with reported studies and to demonstrate that the performance improvements of the proposed mAPO arise from its hybrid search structure rather than from parameter tuning.

### 4.3. Working Mechanism of the Proposed mAPO

The operational structure of the proposed mAPO is summarized in Figure 4, which illustrates how the biological behaviors of APO are combined with the newly incorporated RLS and NM refinements. The algorithm proceeds in an iterative manner, beginning with the initialization of the protozoa population and concluding with the extraction of the global optimum once the maximum iteration limit is reached. At the start of each run, the algorithm generates an initial set of protozoa within the allowable search bounds and assigns the required control parameters. The iteration counter is set to one. The population is then sorted according to fitness, enabling the algorithm to maintain an ordered structure from which the best-performing protozoa can be identified and exploited. Following this sorting stage, each protozoan is evaluated to determine its behavioral path. As shown in Figure 4, this decision divides the update process into two main branches: foraging or dormancy/reproduction.

Under the foraging branch, the protozoan is further classified as autotrophic or heterotrophic, leading to the application of either Equation (10) or Equation (8), respectively. These updates mimic the nutrient-seeking motion of protozoa and account for both long-range exploratory movements and locally informed adjustments. If the protozoan is assigned to the dormancy–reproduction branch, the algorithm determines whether a dormancy update (Equation (12)) or a reproductive update (Equation (13)) is performed. Dormancy introduces diversity by regenerating individuals when necessary, whereas reproduction imitates binary fission by generating new candidate positions around an existing protozoan.

Regardless of the path taken, the modified protozoan position is subsequently evaluated and compared with its previous fitness value. Only improvements are accepted, ensuring that the population evolves monotonically toward more promising regions of the search space. The global best protozoan $X_{\mathrm{best}}$ is then updated, providing a reference point for the enhancement mechanisms introduced in mAPO.

The next stage of the algorithm is governed by the probabilistic condition, rand≤0.5, which determines whether the Nelder–Mead local search or the random learning mechanism will be activated. If the condition is satisfied, the algorithm constructs a simplex around $X_{\mathrm{best}}$ and applies a fixed number of Nelder–Mead iterations, enabling refined exploitation through geometric transformations such as reflection, expansion, and contraction. This process sharpens the search around the most promising region identified by the protozoa. If the condition is not satisfied, three distinct protozoa are randomly selected from the population to trigger the random learning mechanism, which perturbs the individual using population-driven difference information. This mechanism maintains diversity and encourages exploration of alternative search regions, reducing the likelihood of stagnation around deceptive local minima.

After completing the chosen enhancement step, the algorithm examines whether the maximum number of iterations has been reached. If not, the iteration counter is incremented and the entire cycle repeats. When the termination criterion is satisfied, the global optimum and its corresponding fitness value are returned as the final output. Overall, Figure 4 highlights how the proposed mAPO blends APO’s biologically inspired behaviors with stochastic learning and deterministic local refinement. This structured interaction enables the algorithm to achieve a more effective balance between broad exploration and focused exploitation, resulting in improved convergence characteristics and stronger robustness in nonlinear parameter identification tasks.

## 5. Simulation Results

This section presents a detailed performance evaluation of the proposed mAPO using two nonlinear benchmark systems: the chaotic Rössler system [14] and the chaotic permanent magnet synchronous motor [40]. These systems were selected because their nonlinear and potentially chaotic behavior makes parameter identification particularly challenging, offering an appropriate setting for assessing the accuracy, stability, and convergence characteristics of mAPO. For comparison, the algorithm was tested alongside artificial protozoa optimizer (APO) [24], Schrödinger optimizer (SRA) [41], holistic swarm optimization (HSO) [42], elk herd optimizer (EHO) [43] and Young’s double-slit experiment optimizer (YDSE) [44] under identical conditions. Each method was executed for 20 independent runs with a population size (ps) of 30 and a maximum of 400 iterations (${iter}_{max}$). This experimental setup ensures consistent benchmarking and enables a fair statistical comparison across all optimization methods.

The parameter settings adopted in this study were selected to ensure fairness, stability, and reproducibility, while preserving the intrinsic characteristics of the optimization algorithms under comparison. For the APO and the proposed modified version mAPO, the original control parameters governing foraging, dormancy, reproduction, and behavior-switching probabilities were retained exactly as defined in the canonical APO formulation. This choice was made deliberately to maintain the inherent balance between exploration and exploitation embedded in the original algorithm and to ensure that the performance improvements observed in mAPO arise solely from the introduced random learning strategy and the embedded Nelder–Mead simplex refinement, rather than from extensive parameter retuning. The additional parameters associated with the proposed enhancements were determined based on well-established practices in the metaheuristic optimization literature. In particular, the coefficients used in the Nelder–Mead simplex operations (reflection, expansion, and contraction) were selected according to standard recommendations for derivative-free local search, ensuring stable and efficient local refinement. Similarly, the activation probability of the random learning mechanism was chosen to introduce occasional diversity-enhancing perturbations without disrupting the dominant search dynamics of the population. These settings were fixed across all experiments to avoid problem-specific tuning and to preserve generality.

### 5.1. Results on CEC2017 Benchmark Functions

To further assess the generalization capability of the proposed modified artificial protozoa optimizer, additional experiments were conducted using the CEC2017 benchmark function suite, which is widely recognized as a rigorous testbed for evaluating optimization algorithms in high-dimensional and complex search spaces [27]. The benchmark set includes shifted and rotated functions, hybrid functions, and composition functions, each designed to capture different optimization challenges such as nonseparability, variable interaction, and multimodality [45]. All experiments were carried out using a 30-dimensional decision space, a population size of 50, a maximum of 1000 iterations, and 20 independent runs for each function. The statistical performance metrics reported include the best, worst, mean, and standard deviation (SD) of the objective function values.

**Figure 5:**
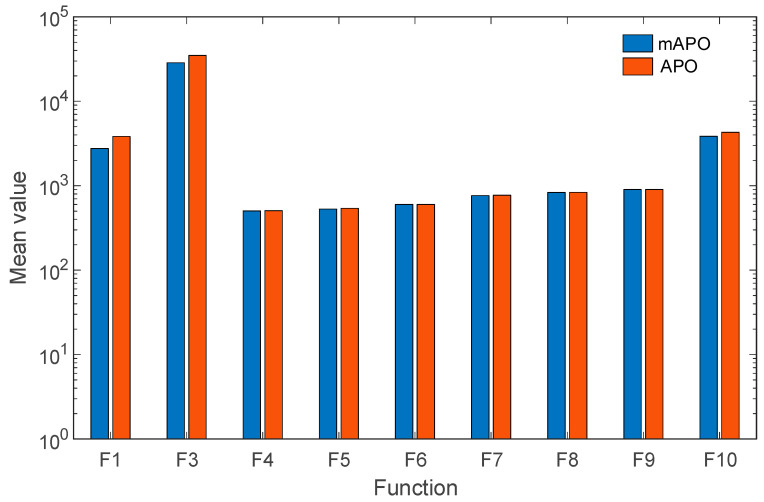
Mean values of CEC2017 shifted and rotated functions for mAPO and APO.

The results for the shifted and rotated functions ($F_{1}$–$F_{10}$), summarized in Table 1, indicate that mAPO consistently attains lower mean objective values than APO across almost all test cases. In addition, reduced standard deviation values are observed for mAPO on several functions, suggesting improved stability and repeatability. Notably, for functions such as $F_{6}$ and $F_{9}$, mAPO reaches the optimal value with near-zero variability, whereas APO exhibits small but non-negligible dispersion. Figure 5 provides a visual representation of the data presented in Table 1.

**Figure 6:**
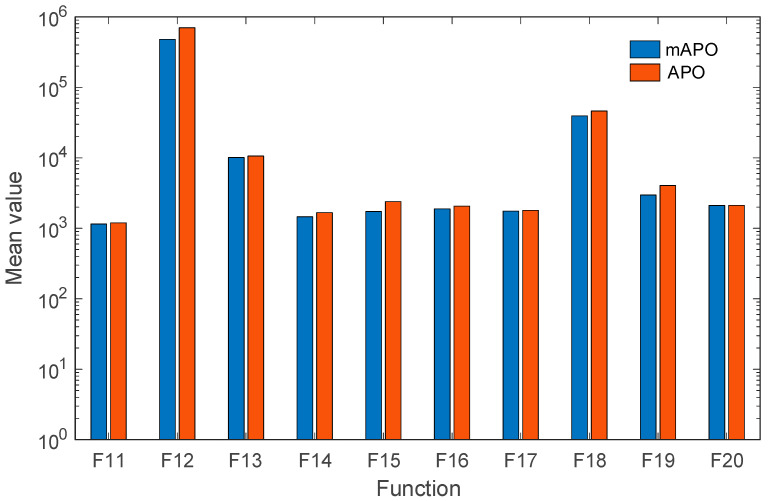
Mean values of CEC2017 hybrid functions for mAPO and APO.

The hybrid function results ($F_{11}$–$F_{20}$), reported in Table 2, further highlight the advantages of the proposed modifications. Hybrid functions are particularly challenging due to their combination of different landscape properties within a single objective. Across this group, mAPO achieves lower mean and worst-case values than APO on most functions, with substantially reduced dispersion for functions such as $F_{14}$, *F*_15_, and $F_{17}$. These outcomes indicate that the proposed random learning and local refinement mechanisms enhance the optimizer’s ability to navigate heterogeneous search spaces. Figure 6 provides a visual representation of the data presented in Table 2.

**Figure 7:**
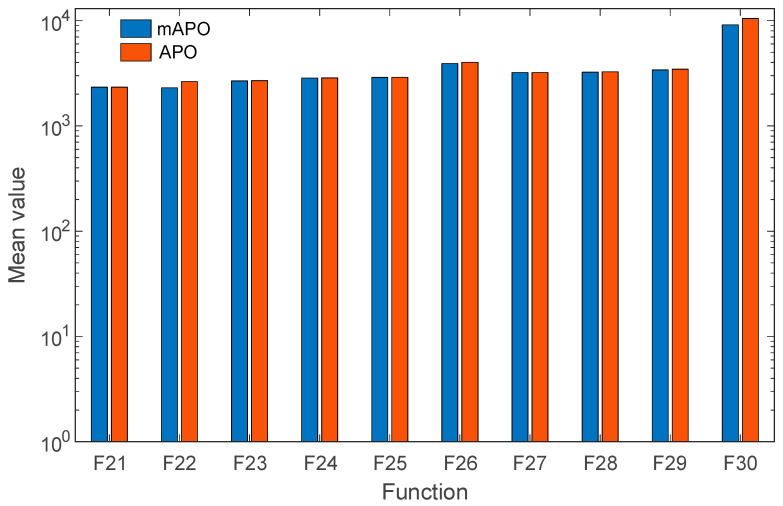
Mean values of CEC2017 composition functions for mAPO and APO.

For the composition functions ($F_{21}$–$F_{30}$), which represent some of the most difficult problems in the CEC2017 suite, the results in Table 3 demonstrate that mAPO maintains a consistent performance advantage over APO. Although both algorithms converge to similar regions of the search space, mAPO generally produces lower mean objective values and smaller standard deviations, particularly for functions $F_{22}$, $F_{26}$, *F*_28_, and $F_{29}$. This behavior suggests that the modified framework provides improved balance between exploration and exploitation in highly irregular landscapes. Figure 7 provides a visual representation of the data presented in Table 3.

Overall, the CEC2017 results confirm that the proposed mAPO algorithm exhibits strong generalization capability in high-dimensional optimization problems. The consistent improvements observed across shifted, hybrid, and composition functions indicate that the proposed modifications are not problem specific, but rather enhance the underlying search dynamics of the algorithm. These findings complement the nonlinear system identification results presented earlier and demonstrate that mAPO is a robust and scalable optimization framework suitable for a wide range of complex optimization tasks

### 5.2. Results of Chaotic Rössler System

#### 5.2.1. Statistical Analysis

The statistical performance of the proposed mAPO on the chaotic Rössler system is summarized in Table 4, where the best, worst, mean, and standard deviation (SD) values of the objective function over 20 independent runs are reported for all competing algorithms. These results provide insight into both the accuracy and the stability of each method. As shown in the table, mAPO achieves the smallest best, worst, and mean objective values among all algorithms, with its best result reaching the extremely low value of $2.9986\times 10^{-32}$. This indicates that mAPO is capable of reaching solutions that reproduce the true system dynamics with negligible error. The corresponding worst value remains several orders of magnitude lower than those of the other optimizers, demonstrating that its performance does not degrade significantly across different runs. The mean objective value also remains on the order of $10^{-32}$, confirming that the algorithm consistently converges toward the true parameters with minimal variation. The standard deviation of mAPO is also the smallest in the comparison, reflecting highly stable convergence behavior. In contrast, the alternative methods (particularly HSO, SRA, and EHO) exhibit noticeably higher mean errors and larger variability across runs. Even the best results obtained by APO and EHO remain several orders of magnitude above those achieved by mAPO, revealing the considerable improvement brought by the proposed modifications. Overall, the statistical findings in Table 4 demonstrate that the incorporation of the random learning mechanism and the Nelder–Mead simplex refinement markedly enhances the accuracy, repeatability, and robustness of APO when applied to chaotic parameter identification.

#### 5.2.2. Change in Objective Function and Estimated Parameters

**Figure 8:**
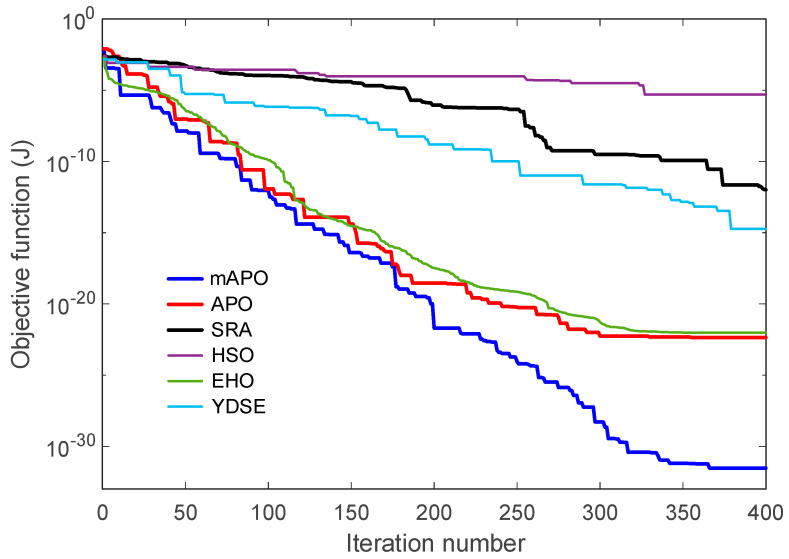
Comparative convergence curves showing the change in objective function value with respect to iteration number for chaotic Rössler system.

The convergence characteristics of the competing algorithms for the chaotic Rössler system are presented in Figure 8, which illustrates how the objective function J evolves with the iteration number. As shown in the figure, the proposed mAPO exhibits the most rapid and stable reduction in the objective value among all tested algorithms. The curve associated with mAPO declines sharply during the initial iterations and continues to decrease in a consistent manner until it reaches values on the order of $10^{-32}$. This behavior demonstrates that the algorithm efficiently identifies the optimal region and maintains steady progress throughout the entire optimization process. In comparison, APO and EHO show moderately fast convergence but settle at higher objective values, several orders of magnitude above those reached by mAPO. The performance of SRA and YDSE is more erratic; both methods show intermittent improvements followed by long periods of stagnation, which suggests difficulty in navigating the highly nonlinear error surface. HSO displays the slowest and least stable convergence behavior, with its curve remaining well above those of the other algorithms. These results collectively indicate that the modified components embedded in mAPO significantly strengthen its ability to cope with the chaotic dynamics of the Rössler system.

**Figure 9:**
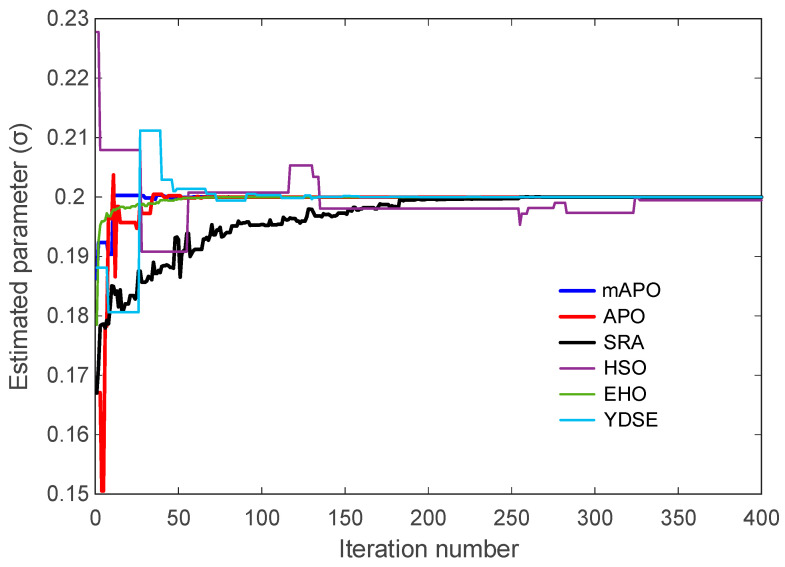
Comparative values of σ parameter with respect to iteration number for chaotic Rössler system.

**Figure 10:**
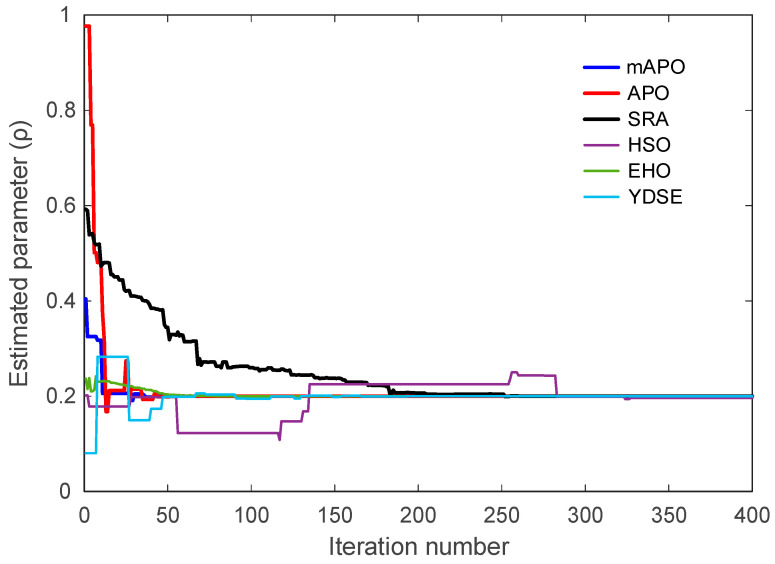
Comparative values of ρ parameter with respect to iteration number for chaotic Rössler system.

**Figure 11:**
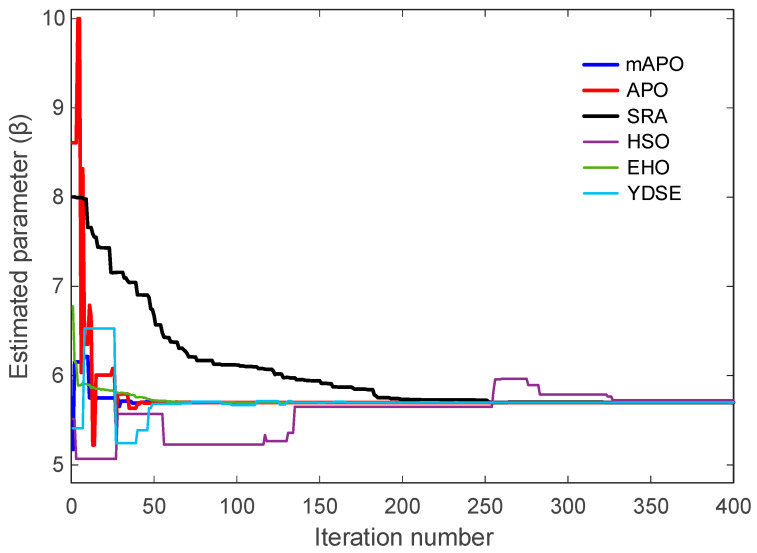
Comparative values of β parameter with respect to iteration number for chaotic Rössler system.

The evolution of the individual system parameters (σ, ρ, and β) is illustrated in Figure 9, Figure 10 and Figure 11. These figures show how each algorithm approaches the true parameter values over the course of the optimization. In Figure 9, which depicts the trajectory of the estimated σ parameter, mAPO reaches the true value rapidly and stabilizes without noticeable oscillation. APO and EHO also approach the correct value, although their early-stage fluctuations are more pronounced. SRA, HSO, and YDSE exhibit substantial variability during the initial iterations, with some trajectories deviating considerably from the true parameter before gradually settling toward a narrow band. The smooth and monotonic convergence achieved by mAPO highlights its robustness in handling the sensitivity associated with the σ component of the Rössler system.

A similar pattern is observed in Figure 10, showing the evolution of the ρ parameter. The mAPO curve quickly aligns with the correct parameter value and maintains this alignment throughout the remaining iterations. APO and EHO display relatively accurate but slower convergence, while SRA, HSO, and YDSE show wider fluctuations and delayed stabilization. In several cases, these methods momentarily converge to incorrect intermediate values before shifting toward the true parameter. The consistent trajectory produced by mAPO again reflects its superior stability and tracking capability.

The evolution of β is presented in Figure 11. The mAPO trajectory reaches the true value early in the optimization process and demonstrates excellent numerical steadiness thereafter. APO and EHO also approximate the true β parameter with reasonable accuracy, though their curves contain small deviations before stabilizing. By contrast, SRA and HSO exhibit substantial early divergence, and their convergence toward the optimal value occurs much later. YDSE shows scattered behavior during the early iterations, further confirming its limited robustness in this setting. These observations reinforce the advantage of mAPO in accurately estimating parameters even when the system exhibits strong nonlinear coupling.

The final identification results are summarized in Table 5, which lists the best objective function values and corresponding parameter estimates obtained by each algorithm. The proposed mAPO achieves the best performance across all metrics, with an objective value of $J=2.9986\times 10^{-32},$ which is far smaller than the results produced by the other algorithms. Moreover, the estimated parameters from mAPO (σ=0.200000000000, ρ=0.200000000000, β=5.700000000000) match the true parameter values with full numerical precision. APO and EHO also obtain parameter estimates near the true values but achieve objective values on the order of $10^{-23}$, confirming that their solutions remain several magnitudes less accurate than those of mAPO. SRA, HSO, and YDSE show more noticeable deviations from the true parameter set, which is consistent with the irregularities observed in their convergence curves.

Taken together, Figure 8, Figure 9, Figure 10 and Figure 11, and Table 5 demonstrate that mAPO consistently outperforms the other algorithms in both convergence speed and parameter estimation accuracy. The sharply declining objective curve, stable parameter trajectories, and precise final estimates confirm that the hybrid modifications incorporated into mAPO greatly enhance its capacity to handle the chaotic and nonlinear identification challenges presented by the Rössler system.

#### 5.2.3. Accurate Parameter Identification

To further evaluate the precision of the estimated parameters, the relative identification error for each algorithm was calculated using the error-rate expression given in Equation (21):

(21) $$Error\;rate=\frac{\left|Actual\;value-Estimated\;value\right|}{Actual\;value}\times 100$$

This metric quantifies the deviation between the true parameter and the estimated value as a percentage, allowing a direct comparison of the identification accuracy achieved by different optimization algorithms. The resulting error rates for the Rössler system parameters σ, ρ, and β are presented in Table 6. As shown in the table, the proposed mAPO achieves exceptionally small error rates across all parameters, reaching values on the order of $10^{-14}$ for both σ and ρ, and producing zero error for β. These results confirm that mAPO reconstructs the true parameter set with essentially full numerical precision. APO and EHO also demonstrate relatively low error rates, though they remain several orders of magnitude larger than those obtained with mAPO. Their estimated values are close to the true parameters but still reflect the small inaccuracies observed earlier in their convergence trajectories. The remaining algorithms (SRA, HSO, and YDSE) exhibit noticeably higher error rates. In particular, HSO displays substantial deviations, reaching error levels exceeding 1% for ρ and nearly 0.45% for β.

These results are consistent with the irregular and slow convergence behavior previously observed for HSO in Figure 8, Figure 9, Figure 10 and Figure 11. SRA and YDSE achieve moderately better accuracy but still fall short of the performance delivered by APO, EHO, and especially mAPO. Overall, the error-rate analysis clearly demonstrates that the proposed mAPO provides the most accurate parameter identification among the compared algorithms. Its ability to yield near-zero error for all parameters confirms that the algorithm not only converges rapidly but also reaches the true optimal solution with remarkable precision. This high degree of accuracy reinforces the value of the integrated random learning and Nelder–Mead refinement mechanisms, which enhance both the reliability and robustness of the identification process.

#### 5.2.4. Comparison with Reported Works

To position the effectiveness of the proposed mAPO within the broader literature, its performance was compared with several reported parameter identification approaches commonly applied to chaotic systems. The selected reference algorithms (flower pollination algorithm (FPA) [30], improved fruit fly algorithm (IFFO) [30], quantum particle swarm optimization (QPSO) [30]) have each been utilized in earlier studies for estimating the parameters of nonlinear and chaotic dynamical models. Their best reported results for the chaotic Rössler system are summarized alongside those of mAPO in Table 7.

As shown in the table, all compared methods successfully converge to the correct parameter values $\left(\sigma =0.2000,\;\rho =0.2000,\;\beta =5.7000\right)$. However, a clear distinction emerges in terms of the achieved objective function values. The proposed mAPO attains an exceptionally small objective value of $J=2.9986\times 10^{-32},$ which is several orders of magnitude smaller than those produced by the reported algorithms. The next best results are obtained by QPSO, with an error value of $2.1341\times 10^{-11}$, followed by IFFO and FPA, which reach values on the order of $10^{-7}$. These numbers indicate that, although the competing methods succeed in identifying parameters that qualitatively reproduce the system dynamics, they do not achieve the extremely low reconstruction error exhibited by mAPO. The superior precision demonstrated by mAPO can be attributed to the integration of the random learning mechanism and the Nelder–Mead simplex refinement, both of which enhance the optimizer’s ability to navigate the Rössler system’s sensitive and highly nonlinear parameter landscape. The comparison confirms that mAPO not only matches but significantly surpasses the best results previously reported in the literature. Its ability to recover the true parameters with full numerical accuracy and minimal objective error highlights the strength of the proposed modifications and positions mAPO as a highly robust tool for identifying chaotic system parameters.

#### 5.2.5. Online Parameter Identification

**Figure 12:**
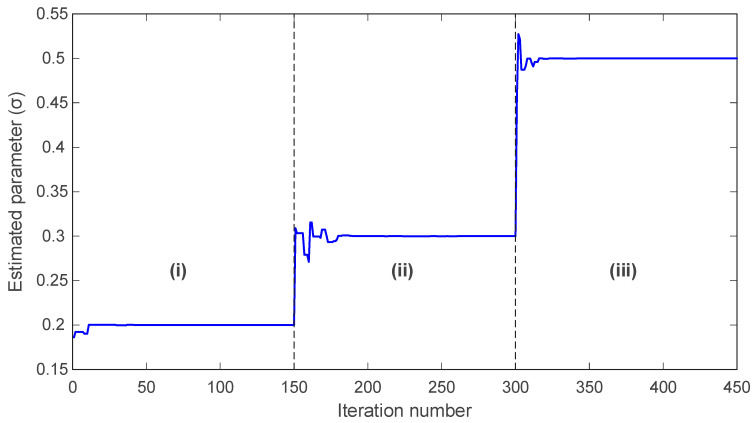
Online identification process of σ parameter for Rössler system.

**Figure 13:**
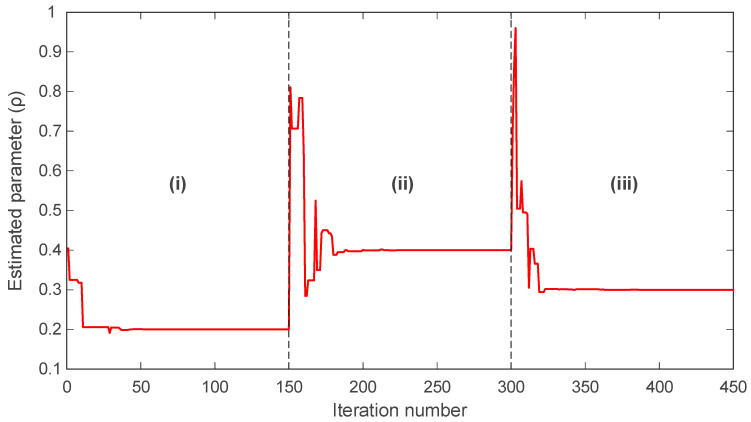
Online identification process of ρ parameter for Rössler system.

**Figure 14:**
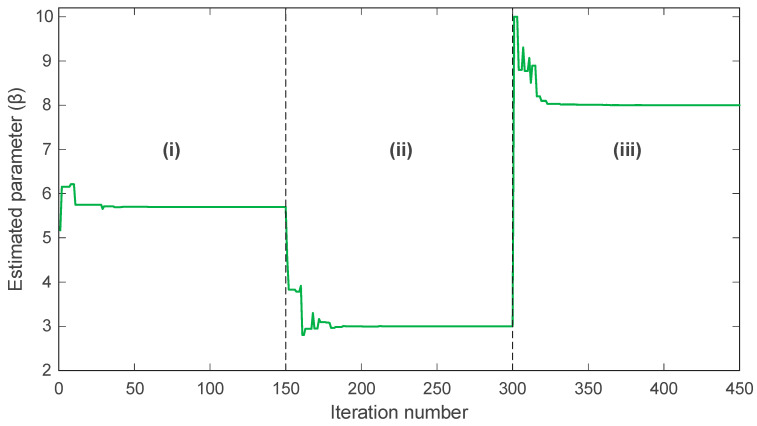
Online identification process of β parameter for Rössler system.

In many practical scenarios, the parameters of chaotic systems do not remain constant and may change abruptly due to external influences or shifts in operating conditions. For this reason, the online identification capability of the proposed mAPO was evaluated by allowing the parameters of the Rössler system to vary across three distinct segments of the simulation. The evolution of the estimated parameters σ, ρ, and β over time is presented in Figure 12, Figure 13 and Figure 14, where each figure is divided into three intervals, denoted as (i), (ii), and (iii), corresponding to different sets of true parameter values.

In Figure 12, the trajectory of the estimated σ parameter is shown. During interval (i), the algorithm converges smoothly to the correct value σ=0.2 and maintains this value with negligible fluctuation. When the true parameter changes to σ=0.3 at the start of interval (ii), the algorithm rapidly detects the shift and adjusts the estimate accordingly, stabilizing close to the new value. A similar behavior is observed at the transition to interval (iii), where the true parameter becomes σ=0.5; mAPO again adapts promptly and converges toward the updated value.

The evolution of ρ, shown in Figure 13, follows the same pattern. The algorithm maintains accurate tracking in interval (i) with ρ=0.2. After the change to ρ=0.4 in interval (ii), the estimate rises rapidly and stabilizes around the new value. When the parameter returns to ρ=0.3 in interval (iii), mAPO responds immediately and adjusts its estimate with minimal overshoot. These transitions demonstrate the algorithm’s ability to detect changes and re-optimize efficiently without sacrificing numerical stability.

The behavior of the β parameter, illustrated in Figure 14, further confirms this capability. In interval (i), the estimate converges to β=5.7. Upon shifting to β=3 in interval (ii), the algorithm corrects its estimate and stabilizes near the new value. When β changes again to 8 in interval (iii), mAPO rapidly adapts and converges to the correct level with only minor transient fluctuations.

A quantitative summary of the actual and estimated parameters in each interval is provided in Table 8. For all three parameters, the estimated values closely match the true values in each segment. The accuracy achieved across all intervals confirms that mAPO reliably tracks parameter variations in real time, even when abrupt changes occur. This performance can be attributed to the algorithm’s integrated learning mechanisms, which enable continuous adaptation while preserving convergence stability. Overall, the results presented in Figure 12, Figure 13 and Figure 14 and Table 8 demonstrate that the proposed mAPO framework is highly effective for online parameter identification. Its capacity to detect changing system dynamics and promptly re-estimate parameters makes it particularly suitable for chaotic systems, where sensitivity to initial conditions and parameter variability requires robust, rapid, and stable identification methods.

### 5.3. Results of PMSM System

#### 5.3.1. Statistical Analysis

The statistical performance of the proposed mAPO on the chaotic PMSM system is summarized in Table 9, where the best, worst, mean, and standard deviation values of the objective function obtained from 20 independent runs are presented for all competing algorithms. These results provide an overall assessment of the accuracy, robustness, and consistency exhibited by each optimization method. As shown in the table, mAPO achieves perfect statistical performance, obtaining zero error across all four indicators. Both the best and worst objective values recorded by mAPO are exactly zero, demonstrating that the algorithm not only reaches the globally optimal solution but does so reliably across every run. The mean and SD values are also zero, confirming the complete absence of performance variability. This consistency indicates that the proposed enhancements enable mAPO to handle the nonlinear and potentially chaotic structure of the PMSM model with exceptional reliability. In comparison, APO produces highly competitive results, with its best objective value reaching $1.4030\times 10^{-26}$, though still several orders of magnitude above the exact zero achieved by mAPO. The small but nonzero SD value associated with APO suggests minor variation between runs. The remaining algorithms (SRA, HSO, EHO, and YDSE) show significantly greater deviations. SRA and YDSE exhibit performance in the range of $10^{-16}$ to $10^{-19}$ for the best values, but their worst and mean values reveal that consistency is lacking. HSO and EHO show the weakest performance among the group, with best values on the order of $10^{-10}$ and $10^{-11}$, respectively, and considerably larger worst-case and SD values. These results reflect greater sensitivity to initial population distribution, insufficient exploitation strength, and a tendency to stagnate in locally optimal regions of the PMSM parameter space. Overall, the statistical findings in Table 9 clearly demonstrate the superior robustness of mAPO for PMSM parameter identification. Its ability to return an exact zero objective value across all runs highlights both the algorithm’s high convergence precision and its immunity to stochastic variability. These results confirm that the integration of the random learning strategy and the Nelder–Mead refinement enables mAPO to outperform the benchmark algorithms by a substantial margin when applied to the complex PMSM system.

#### 5.3.2. Change in Objective Function and Estimated Parameters

**Figure 15:**
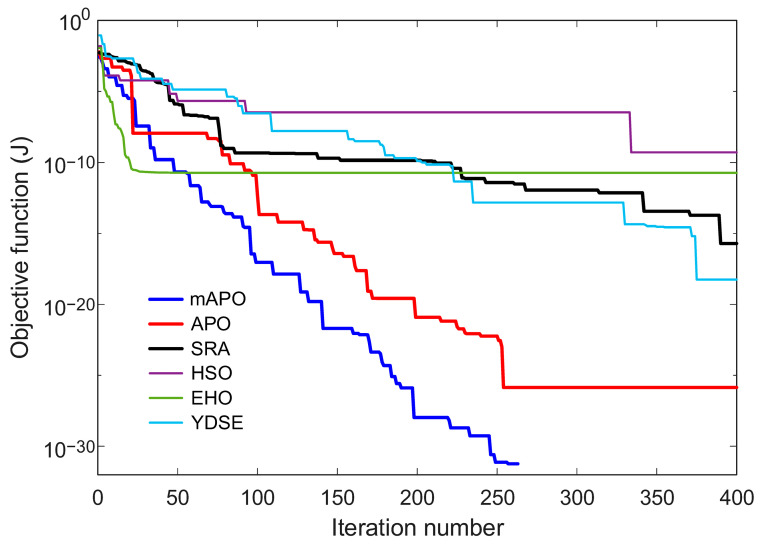
Comparative convergence curves showing the change in objective function value with respect to iteration number for PMSM system.

The convergence characteristics of the competing algorithms for the PMSM system are illustrated in Figure 15, which presents the evolution of the objective function J over 400 iterations. As seen in the figure, the proposed mAPO demonstrates a markedly faster and more stable decline in the objective value compared with all other algorithms. The convergence curve for mAPO drops sharply during the initial stage and continues decreasing monotonically until it reaches an exact zero value, indicating perfect reconstruction of the PMSM dynamics. APO and EHO also display relatively good convergence trends, but their curves level off at nonzero objective values several orders of magnitude higher than those achieved by mAPO. In contrast, SRA, HSO, and YDSE converge more slowly and exhibit visible fluctuations, suggesting difficulty in navigating the complex nonlinear error landscape of the PMSM model.

**Figure 16:**
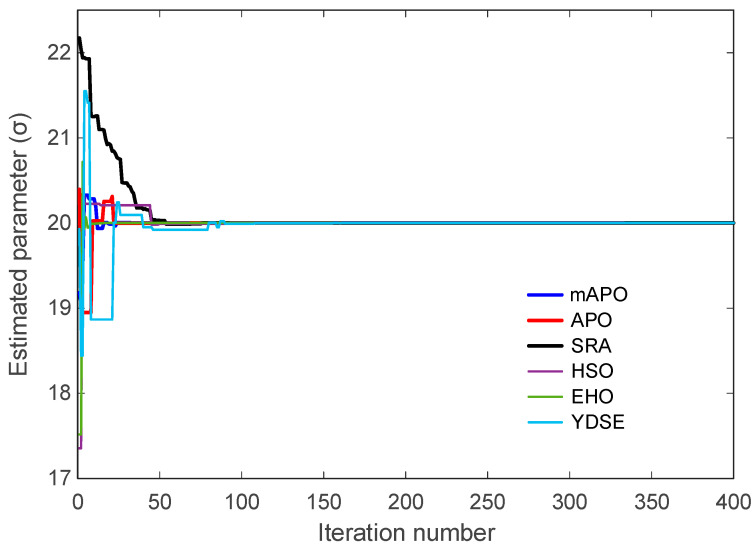
Comparative values of σ parameter with respect to iteration number for PMSM system.

**Figure 17:**
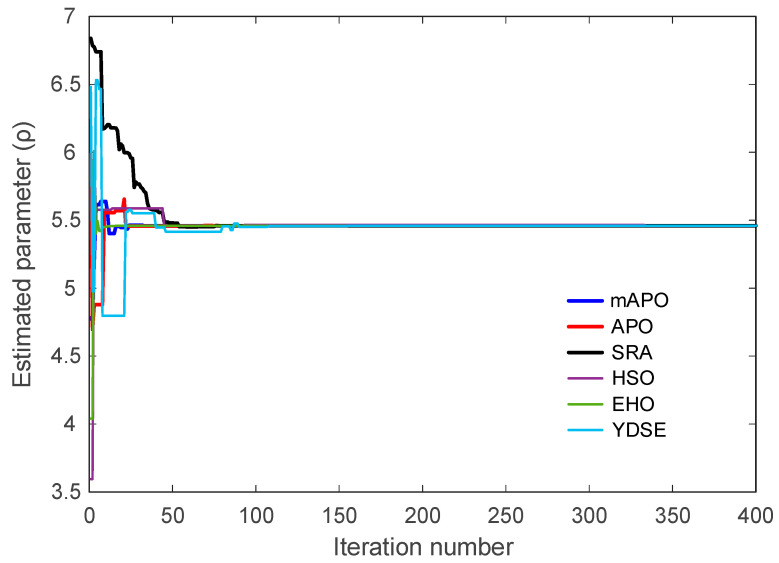
Comparative values of ρ parameter with respect to iteration number for PMSM system.

The evolution of the estimated PMSM parameters σ and ρ is shown in Figure 16 and Figure 17, respectively. In both figures, mAPO converges rapidly to the true parameter values and maintains exceptional stability throughout the simulation. The estimated σ value produced by mAPO aligns almost immediately with the true value of 20, while the ρ estimate stabilizes at 5.46 with similarly negligible deviation. The smoothness of the mAPO trajectories indicates that the algorithm effectively balances exploration and exploitation when handling the nonlinear PMSM parameter space. The remaining algorithms display noticeably less stable behavior. APO and EHO converge near the true parameter values but show small oscillations before settling. SRA and YDSE exhibit more pronounced fluctuations in the early iterations, requiring additional time to approach the correct values. HSO demonstrates the weakest performance, deviating significantly from the true values during early iterations and converging only slowly and with residual error. These observations are consistent with the convergence patterns in Figure 15 and emphasize the advantage of the hybrid mechanisms integrated into mAPO.

The final identification results for all algorithms are summarized in Table 10, which lists the best objective values and the corresponding optimal parameter estimates. As shown, mAPO uniquely achieves an exact objective value of zero, while simultaneously recovering the true parameters with complete numerical accuracy: σ=20.000000000000, ρ=5.460000000000. APO and EHO also produce accurate parameter estimates but do not achieve error-free objective values, instead converging to $1.4030\times 10^{-26}$ and $1.8692\times 10^{-11}$, respectively. SRA, YDSE, and HSO exhibit progressively larger objective values and slightly less accurate parameter estimates. Notably, HSO yields the least precise results, as reflected by its larger deviations in both σ and ρ. Collectively, Figure 15, Figure 16 and Figure 17, and Table 10 demonstrate that mAPO provides the most reliable and accurate parameter identification performance for the PMSM system. Its rapid convergence, stable parameter trajectories, and exact reconstruction of the true parameters confirm that the incorporation of the random learning and Nelder–Mead refinement mechanisms significantly enhances the optimizer’s capability when dealing with nonlinear and chaotic electromechanical systems.

#### 5.3.3. Accurate Parameter Identification

To assess the precision of the estimated PMSM parameters, a quantitative error analysis was conducted using the relative error expression provided earlier in Equation (21). This metric evaluates the deviation between the true parameter value and its corresponding estimate obtained through each optimization method, thereby allowing a direct comparison of identification accuracy across competing algorithms. The resulting error percentages for the PMSM parameters σ and ρ are summarized in Table 11.

The results clearly show that the proposed mAPO achieves perfect identification accuracy, yielding zero error for both parameters. This outcome confirms that every run of mAPO converged exactly to the true system parameters, a finding consistent with the zero-valued best, worst, mean, and standard deviation statistics presented earlier in Table 9. The ability of mAPO to recover the parameters with complete numerical precision demonstrates the effectiveness of its integrated learning mechanisms and the synergy between the random learning and Nelder–Mead refinement stages, which collectively enhance both global search capacity and fine-scale exploitation. APO also performs at a high accuracy level, achieving error rates on the order of $10^{-12}$ for both parameters. Although these values are extremely small, they remain several orders of magnitude above the exact-zero accuracy produced by mAPO. This difference reflects the added refinement capability incorporated into the modified algorithm. SRA and YDSE exhibit moderate accuracy, with error values ranging from $10^{-6}$ to $10^{-7}$. These results indicate that both algorithms can approximate the true parameters reliably but lack the robustness needed for consistently achieving near-zero numerical error. In contrast, HSO and EHO show noticeably larger deviations, with error levels reaching as high as $10^{-3}$. Their performance suggests weaker convergence stability and a tendency to become influenced by the nonlinearities inherent in the PMSM system. Overall, the comparison in Table 11 confirms that mAPO provides the most accurate parameter estimation among the considered algorithms. Its ability to maintain zero error for both σ and ρ underscores the algorithm’s strong numerical stability and its capability to fully reconstruct the PMSM model. This high level of accuracy further validates the modifications introduced into APO and highlights mAPO as a reliable and highly precise framework for nonlinear parameter identification, especially in systems characterized by chaotic or strongly coupled dynamics.

#### 5.3.4. Comparison with Reported Works

To further contextualize the identification capability of the proposed mAPO, its performance was compared with several optimization approaches previously reported in the PMSM parameter identification literature. The selected reference methods include the improved Lozi map-based chaotic optimization algorithm (ILCOA) [33], hybrid differential evolution algorithm and artificial bee colony algorithm (DE/ABC) [33], genetic algorithm (GA) [33]. These algorithms have been applied in earlier studies for estimating the PMSM parameters and therefore offer an appropriate external benchmark for evaluating the accuracy achieved by mAPO.

The comparative results are presented in Table 12, which lists the best objective values together with the corresponding parameter estimates. As shown in the table, mAPO is the only algorithm that achieves a perfect objective value of 0, indicating complete reconstruction of the PMSM dynamics without any residual modeling error. ILCOA and DE/ABC also succeed in reaching extremely small objective values (on the order of $10^{-30}$ and $10^{-27}$, respectively) and correctly identify the parameters σ=20.0000 and ρ=5.4600. Although their estimates match the true parameters, the presence of a nonzero objective value suggests a minute but detectable discrepancy in the reconstructed trajectory. The GA exhibits the weakest performance among the compared methods, producing a noticeably higher objective value of 0.0180, accompanied by small deviations in the recovered parameters (σ=19.9593 and ρ=5.4749). These deviations confirm that GA is less capable of navigating the nonlinear PMSM parameter landscape and struggles to attain the same level of precision achieved by the other approaches.

Overall, the comparison in Table 12 clearly demonstrates that the proposed mAPO outperforms all reported methods. While ILCOA and DE/ABC deliver highly accurate solutions, mAPO distinguishes itself by achieving zero reconstruction error and recovering the true PMSM parameters with complete numerical fidelity. This superior performance reflects the combined strength of the random learning mechanism and the embedded Nelder–Mead refinement, which together provide a balanced and highly effective search process. The results thereby position mAPO as a state-of-the-art tool for parameter identification in nonlinear and chaotic electromechanical systems such as the PMSM.

#### 5.3.5. Online Parameter Identification

To further evaluate the adaptability of the proposed mAPO, an online identification experiment was carried out in which the PMSM parameters σ and ρ were allowed to change abruptly during the simulation. This setup emulates realistic operating conditions where motor behavior may shift due to load variations, parameter drift, or external disturbances. The true parameter values were intentionally divided into three distinct intervals, denoted as parts (i), (ii), and (iii). In each interval, new values of σ and ρ were assigned, and the algorithm was required to detect these changes and rapidly converge to the updated values.

**Figure 18:**
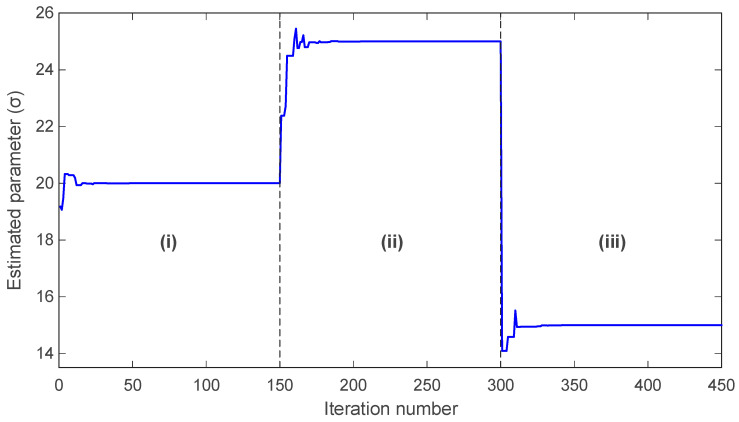
Online identification process of σ parameter for PMSM system.

The evolution of the estimated σ parameter is presented in Figure 18. During part (i), the algorithm converges smoothly to the correct value of σ=20 and maintains a stable estimate with negligible fluctuation. When the true parameter shifts to σ=25 at the beginning of part (ii), mAPO responds immediately and adjusts its estimate toward the new value with a short transient phase. At the transition to part (iii), where the true parameter decreases to σ=15, the algorithm again detects the change promptly and converges rapidly to the correct value. Throughout all three segments, the estimation trajectory remains stable, showing no overshoot or oscillatory behavior that would suggest susceptibility to abrupt parameter variations.

**Figure 19:**
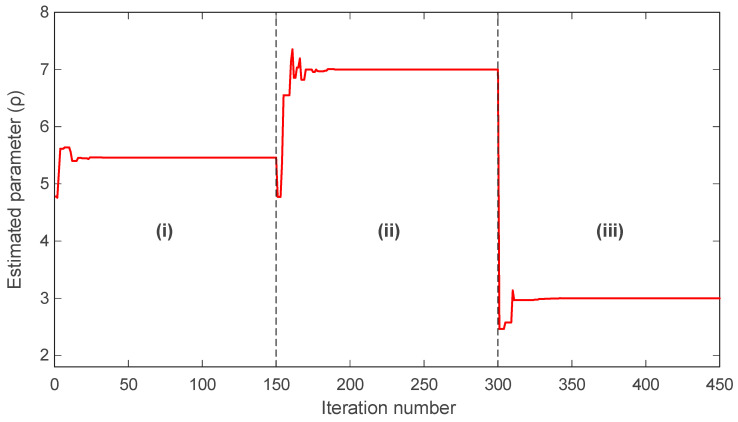
Online identification process of ρ parameter for PMSM system.

A similar trend is observed in the estimation of the ρ parameter, shown in Figure 19. In part (i), mAPO correctly identifies ρ=5.46 and sustains a steady estimate. Once the parameter changes to ρ=7 in part (ii), the algorithm quickly adapts and stabilizes near the new value. Finally, when ρ is reduced to 3 during part (iii), mAPO again achieves rapid convergence, with only a brief and well-damped transient adjustment. The consistency of these transitions indicates that the algorithm maintains a balanced exploitation–exploration behavior even under dynamic conditions.

A numerical summary of the actual and estimated values for each interval is provided in Table 13. The results confirm that mAPO reproduces the true parameter values with complete accuracy across all three parts. No deviation is observed between the actual and estimated values of either parameter, which aligns with the perfectly convergent behavior shown in the figures. This accuracy demonstrates that mAPO is capable not only of identifying static PMSM parameters with high precision but also of tracking time-varying dynamics without degradation in performance. Overall, the findings of Figure 18 and Figure 19 and Table 13 validate the strong online identification capability of the proposed mAPO. The rapid convergence following each parameter shift, combined with the absence of overshoot or drift, highlights the robustness of the integrated random learning and Nelder–Mead refinement mechanisms in managing abrupt changes in nonlinear electromechanical systems. Such responsiveness is particularly important in applications where real-time parameter monitoring is essential for maintaining stability and performance.

### 5.4. Discussion

The characterization of the obtained solutions as promising is grounded in the quantitative evidence reported throughout this study. From a general optimization perspective, the evaluation on the CEC2017 benchmark suite demonstrates that the proposed mAPO consistently yields lower mean objective values and reduced standard deviations across shifted, hybrid, and composition functions when compared with the original APO. This behavior is particularly evident for challenging benchmark functions such as $F_{12}$, $F_{14}$, $F_{15}$, $F_{22}$, and $F_{26}$, where mAPO exhibits both improved solution quality and enhanced statistical reliability across independent runs. These numerical indicators confirm that the observed performance improvements are systematic rather than incidental.

The same conclusion is reinforced by the nonlinear system identification studies. For the chaotic Rössler system, objective function values on the order of 10^−32^ are obtained using mAPO, with corresponding parameter estimation errors reaching 10^−14^ or vanishing entirely. In contrast, competing algorithms converge to significantly higher residual error levels. For the PMSM system, exact reconstruction is achieved, with zero objective value, zero standard deviation, and zero parameter error across all runs. These outcomes demonstrate that the identified parameter sets reproduce the underlying system dynamics with full numerical precision. Accordingly, the term promising solutions is used here to denote solutions that are supported by measurable reductions in modeling error, improved convergence stability, and consistent statistical performance, rather than by qualitative observation alone.

A more detailed examination of the Rössler benchmark further clarifies the behavior of the proposed optimizer. The statistical results reported in Table 4 show that the best, worst, and mean values of the objective function obtained by mAPO are several orders of magnitude smaller than those achieved by the competing optimizers, while the associated standard deviation remains minimal. This indicates not only high accuracy but also strong repeatability across independent runs. The convergence curves in Figure 8 corroborate this observation, showing a rapid and monotonic decrease toward very small objective values for mAPO, whereas alternative methods either stagnate or exhibit irregular convergence patterns. In addition, the parameter evolution plots in Figure 9, Figure 10 and Figure 11 reveal that the estimates of σ, ρ, and β converge rapidly to their true values and remain stable thereafter. These trends are quantified in Table 5 and Table 6, where mAPO attains near-exact numerical agreement with the actual parameters, with error rates down to the order of 10^−14^ or zero.

When the Rössler system is considered in relation to previously reported methods, the advantages of the modified scheme become more evident. As shown in Table 7, all compared approaches converge to the correct parameter triplet [σ, ρ, β] = [0.2,0.2,5.7]. However, the objective value achieved by mAPO is several orders of magnitude smaller than those reported for FPA, IFFO, and QPSO. This implies that, although competing algorithms can reproduce the qualitative structure of the chaotic attractor, a small but persistent mismatch remains in their reconstructed trajectories. In contrast, mAPO effectively eliminates this discrepancy, indicating that the integration of random learning and Nelder–Mead simplex refinement enables more efficient exploitation of promising regions and more precise solution refinement.

The online identification results for the Rössler system further highlight the adaptability of the proposed framework. By introducing piecewise changes in σ, ρ, and β, the system parameters are forced to transition between distinct operating regimes. The time histories presented in Figure 12, Figure 13 and Figure 14 show that mAPO tracks each abrupt parameter change with short transients and without noticeable overshoot, before settling rapidly at the new values. The agreement between actual and estimated parameters reported in Table 8 confirms the absence of steady-state bias. These findings indicate that the combination of global exploration, random learning, and local simplex-based refinement provides sufficient flexibility for the optimizer to escape a previously converged region and re-identify parameters when the system dynamics change.

A similar but more demanding scenario is observed for the PMSM system, which involves nonlinear electromechanical coupling and potential chaotic behavior. In this case, mAPO achieves perfect statistical performance, with the best, worst, mean, and standard deviation of the objective function all equal to zero, as reported in Table 9. While APO produces very small but nonzero errors, SRA, HSO, EHO, and YDSE exhibit noticeably larger objective values and higher variability. The convergence curves in Figure 15 and the parameter trajectories in Figure 16 and Figure 17 are consistent with these statistics, showing that mAPO reaches the true parameters σ=20 and ρ=5.46 within a few iterations and remains stable thereafter. These observations are confirmed by Table 10 and Table 11, where mAPO reconstructs the PMSM parameters with zero error, whereas alternative methods yield nonzero estimation errors ranging from very small to clearly significant.

Additional insight is provided by the comparison with PMSM identification approaches reported in the literature. As shown in Table 12, ILCOA and DE/ABC are capable of achieving very small objective values and matching the true parameter values; however, their residual errors remain above zero. The GA-based solution exhibits both a relatively large objective value and small parameter deviations. In contrast, mAPO attains a zero objective value while exactly recovering the true parameters, implying that the reconstructed PMSM trajectory is indistinguishable from the reference data within numerical precision. This outcome suggests that the hybrid search structure adopted in mAPO achieves a more favorable balance between exploration and exploitation than those employed in the comparative methods.

The online PMSM identification experiment reinforces this conclusion. When σ and ρ are forced to change across three consecutive intervals, the responses shown in Figure 18 and Figure 19 demonstrate that mAPO responds promptly to each parameter variation and converges to the new values with minimal transient distortion. As confirmed by Table 13, the final estimates coincide exactly with the actual parameters in all segments. From a practical perspective, this property is particularly relevant for applications such as adaptive control, fault diagnosis, and health monitoring, where system parameters may vary due to ageing, saturation effects, or external disturbances.

Taken together, the results highlight several key characteristics of the proposed mAPO framework. First, the incorporation of the random learning mechanism enhances global exploration and reduces the risk of premature convergence, as reflected by consistently lower objective values and smoother convergence behavior. Second, the embedded Nelder–Mead simplex refinement enables efficient local exploitation around high-quality candidate solutions, explaining the extremely small final errors and the zero-error statistics observed for the PMSM system. Third, the algorithm demonstrates strong robustness and adaptability, maintaining high identification accuracy under both static and time-varying conditions in chaotic environments.

At the same time, certain aspects warrant further investigation. Although excellent performance has been demonstrated for two benchmark systems using fixed population sizes and iteration limits, the sensitivity of mAPO to its internal control parameters has not yet been systematically examined. In addition, the computational overhead introduced by the random learning and simplex refinement stages has not been explicitly quantified. For large-scale or real-time applications, a more detailed analysis of computational cost and execution time would therefore be beneficial. Future work may also consider extensions to higher-dimensional systems and experimentally measured data, where measurement noise, unmodeled dynamics, and real-time constraints are present.

Finally, it is emphasized that the present work is confined to optimization-based identification of unknown parameters in nonlinear dynamic systems. The use of more than one benchmark model serves solely to evaluate the generality and robustness of the proposed optimizer and does not imply any form of system coupling, fusion, or combined modeling. Each system is treated independently, with its own mathematical formulation, parameter set, and objective function. Accordingly, the proposed framework should be interpreted strictly as an optimization process applied to parameter identification problems, and the scope of the study remains limited to simulation-based evaluation under controlled modeling conditions.

## 6. Conclusions

In this study, a modified artificial protozoa optimizer, named mAPO, was developed and applied to both general optimization problems and the parameter identification of nonlinear and chaotic dynamic systems. The proposed approach introduces a multi-strategy enhancement that strengthens the global search capability and local refinement stages of the original APO framework. By incorporating a judicious combination of foraging-based position updates, adaptive dormancy mechanisms, a probabilistic random learning module, and the Nelder–Mead simplex search, the optimizer was endowed with an improved balance between exploration and exploitation. This enhancement constitutes the core novelty of the work, as it enables mAPO to navigate complex, multimodal optimization landscapes with greater reliability, stability, and accuracy than the original APO and several well-known metaheuristic techniques. The effectiveness and generalization capability of the proposed framework were first validated using the CEC2017 benchmark function suite, which comprises shifted, rotated, hybrid, and composition functions designed to challenge optimization algorithms in high-dimensional search spaces. Across these benchmarks, mAPO consistently achieved lower mean objective values and reduced standard deviations compared with the original APO, demonstrating improved robustness and statistical reliability. These results confirmed that the benefits of the proposed hybridization are not restricted to system-specific identification tasks, but extend to general-purpose optimization problems characterized by severe nonlinearity and multimodality. Following this general assessment, the performance of mAPO was evaluated through extensive experiments on two nonlinear benchmark systems: the chaotic Rössler system and a PMSM model. Both systems are recognized for their sensitivity to initial conditions and parameter variations, making them suitable platforms for assessing identification algorithms. Across all comparative studies, mAPO consistently achieved superior results. It reached the lowest objective function values, produced highly accurate parameter estimates, and demonstrated remarkable convergence characteristics. Statistical analyses further revealed that the proposed algorithm exhibited minimal variance across repeated trials, underscoring its robustness and dependable performance. In the Rössler system case, mAPO successfully recovered the true chaotic parameters with negligible error while maintaining significantly faster and more stable convergence than APO, SRA, HSO, EHO, and YDSE. It also outperformed methods reported in earlier studies, such as FPA, IFFO, and QPSO. A similar level of performance was observed for the PMSM system, where mAPO again surpassed contemporary algorithms in terms of estimation accuracy, convergence speed, and statistical consistency. In both offline and online identification scenarios, the optimizer demonstrated an exceptional ability to adapt to abrupt parameter changes, tracking transitions smoothly and without divergence or steady-state bias. Overall, the findings confirm that the proposed mAPO provides a powerful and reliable optimization framework for both high-dimensional benchmark problems and the identification of unknown parameters in nonlinear and chaotic systems. Its enhanced learning and refinement mechanisms enable it to overcome premature convergence, preserve strong global exploration, and achieve fast and precise local refinement. These qualities render mAPO a promising candidate for broader applications, including adaptive control, fault diagnosis, dynamic state estimation, and other engineering problems involving complex optimization landscapes.

## Figures and Tables

**Table 1:** Statistical results of CEC2017 shifted and rotated functions.

Function (Optimal)	Optimizer	Best	Worst	Mean	SD
$F_{1}$ (100)	mAPO	1.0597 × 10^2^	1.1159 × 10^4^	2.7621 × 10^3^	2.9540 × 10^3^
	APO	1.5650 × 10^2^	1.5083 × 10^4^	3.8410 × 10^2^	4.1147 × 10^3^
$F_{3}$ (300)	mAPO	1.6680 × 10^4^	4.2569 × 10^4^	2.8626 × 10^3^	8.3274 × 10^3^
	APO	2.0813 × 10^4^	4.5469 × 10^4^	3.4949 × 10^4^	6.9960 × 10^3^
$F_{4}$ (400)	mAPO	4.3648 × 10^2^	5.3064 × 10^2^	5.0230 × 10^2^	2.2329 × 10^1^
	APO	4.7641 × 10^2^	5.3198 × 10^2^	5.0705 × 10^2^	1.8756 × 10^1^
$F_{5}$ (500)	mAPO	5.2024 × 10^2^	5.3979 × 10^2^	5.2911 × 10^2^	5.9031 × 10^0^
	APO	5.2118 × 10^2^	5.5969 × 10^2^	5.3975 × 10^2^	9.0393 × 10^0^
$F_{6}$ (600)	mAPO	6.0000 × 10^2^	6.0000 × 10^2^	6.0000 × 10^2^	9.6929 × 10^−4^
	APO	6.0000 × 10^2^	6.0023 × 10^2^	6.0002 × 10^2^	5.2518 × 10^−2^
$F_{7}$ (700)	mAPO	7.4914 × 10^2^	7.8367 × 10^2^	7.6434 × 10^2^	7.9024 × 10^0^
	APO	7.5340 × 10^2^	7.8570 × 10^2^	7.7379 × 10^2^	7.3463 × 10^0^
$F_{8}$ (800)	mAPO	8.1691 × 10^2^	8.4154 × 10^2^	8.3095 × 10^2^	5.9594 × 10^0^
	APO	8.2387 × 10^2^	8.4865 × 10^2^	8.3592 × 10^2^	6.6354 × 10^0^
$F_{9}$ (900)	mAPO	9.0000 × 10^2^	9.0470 × 10^2^	9.0212 × 10^2^	1.6971 × 10^0^
	APO	9.0002 × 10^2^	9.1505 × 10^2^	9.0309 × 10^2^	3.2806 × 10^0^
$F_{10}$ (1000)	mAPO	3.0553 × 10^3^	4.4032 × 10^3^	3.8516 × 10^3^	3.9807 × 10^2^
	APO	3.7673 × 10^3^	4.7880 × 10^2^	4.2870 × 10^3^	3.1524 × 10^2^

**Table 2:** Statistical results of CEC2017 hybrid functions.

Function (Optimal)	Optimizer	Best	Worst	Mean	SD
$F_{11}$ (1100)	mAPO	1.1232 × 10^3^	1.1757 × 10^3^	1.1493 × 10^3^	2.0465 × 10^1^
	APO	1.1492 × 10^3^	1.2339 × 10^3^	1.1922 × 10^3^	2.3343 × 10^1^
$F_{12}$ (1200)	mAPO	2.2050 × 10^4^	1.3267 × 10^6^	4.7905 × 10^5^	4.4392 × 10^5^
	APO	5.7930 × 10^4^	1.8576 × 10^6^	7.0120 × 10^5^	6.1331 × 10^5^
$F_{13}$ (1300)	mAPO	1.4626 × 10^3^	2.2681 × 10^4^	1.0126 × 10^4^	7.0911 × 10^3^
	APO	2.0920 × 10^3^	4.1325 × 10^4^	1.0626 × 10^4^	1.1837 × 10^4^
$F_{14}$ (1400)	mAPO	1.4312 × 10^3^	1.5096 × 10^3^	1.4549 × 10^3^	1.9696 × 10^1^
	APO	1.4428 × 10^3^	5.0281 × 10^3^	1.6656 × 10^3^	7.9460 × 10^2^
$F_{15}$ (1500)	mAPO	1.5367 × 10^3^	2.2302 × 10^3^	1.7301 × 10^3^	1.7522 × 10^2^
	APO	1.6290 × 10^3^	5.6924 × 10^3^	2.3900 × 10^3^	1.0470 × 10^3^
$F_{16}$ (1600)	mAPO	1.6866 × 10^3^	2.0499 × 10^3^	1.8855 × 10^3^	9.8810 × 10^1^
	APO	1.7902 × 10^3^	2.2630 × 10^3^	2.0653 × 10^3^	1.3108 × 10^2^
$F_{17}$ (1700)	mAPO	1.7252 × 10^3^	1.7782 × 10^3^	1.7524 × 10^3^	1.2701 × 10^1^
	APO	1.7357 × 10^3^	1.8972 × 10^3^	1.7947 × 10^3^	5.2176 × 10^1^
$F_{18}$ (1800)	mAPO	3.4756 × 10^3^	7.7173 × 10^4^	3.9441 × 10^4^	1.9785 × 10^4^
	APO	7.3455 × 10^3^	9.1656 × 10^4^	4.6232 × 10^4^	2.1748 × 10^4^
$F_{19}$ (1900)	mAPO	1.9432 × 10^3^	1.3595 × 10^4^	2.9631 × 10^3^	2.5964 × 10^3^
	APO	1.9599 × 10^3^	1.6961 × 10^4^	4.0404 × 10^3^	3.7084 × 10^3^
$F_{20}$ (2000)	mAPO	2.0390 × 10^3^	2.2322 × 10^3^	2.1056 × 10^3^	5.8442 × 10^1^
	APO	2.0547 × 10^3^	2.2077 × 10^3^	2.1176 × 10^3^	5.4148 × 10^1^

**Table 3:** Statistical results of CEC2017 composition functions.

Function (Optimal)	Optimizer	Best	Worst	Mean	SD
$F_{21}$ (2100)	mAPO	2.3224 × 10^3^	2.3451 × 10^3^	2.3304 × 10^3^	6.9588 × 10^0^
	APO	2.3255 × 10^3^	2.3490 × 10^3^	2.3345 × 10^3^	7.2007 × 10^0^
$F_{22}$ (2200)	mAPO	2.3000 × 10^3^	2.3036 × 10^3^	2.3008 × 10^3^	1.2712 × 10^0^
	APO	2.3001 × 10^3^	5.7087 × 10^3^	2.6340 × 10^3^	1.0226 × 10^3^
$F_{23}$ (2300)	mAPO	2.6598 × 10^3^	2.6889 × 10^3^	2.6748 × 10^3^	7.7671 × 10^0^
	APO	2.6708 × 10^3^	2.7128 × 10^3^	2.6870 × 10^3^	9.1679 × 10^0^
$F_{24}$ (2400)	mAPO	2.8379 × 10^3^	2.8580 × 10^3^	2.8454 × 10^3^	5.1676 × 10^0^
	APO	2.8394 × 10^3^	2.8701 × 10^3^	2.8546 × 10^3^	9.3008 × 10^0^
$F_{25}$ (2500)	mAPO	2.8845 × 10^3^	2.8882 × 10^3^	2.8874 × 10^3^	8.9722 × 10^−1^
	APO	2.8870 × 10^3^	2.9091 × 10^3^	2.8917 × 10^3^	6.5943 × 10^0^
$F_{26}$ (2600)	mAPO	3.7411 × 10^3^	3.9896 × 10^3^	3.9072 × 10^3^	7.6966 × 10^1^
	APO	3.7529 × 10^3^	4.2193 × 10^3^	4.0162 × 10^3^	1.1590 × 10^2^
$F_{27}$ (2700)	mAPO	3.1997 × 10^3^	3.2107 × 10^3^	3.2064 × 10^3^	3.7247 × 10^0^
	APO	3.2013 × 10^3^	3.2283 × 10^3^	3.2122 × 10^3^	6.1840 × 10^0^
$F_{28}$ (2800)	mAPO	3.2043 × 10^3^	3.2622 × 10^3^	3.2353 × 10^3^	1.9119 × 10^1^
	APO	3.2111 × 10^3^	3.3367 × 10^3^	3.2596 × 10^3^	2.9520 × 10^1^
$F_{29}$ (2900)	mAPO	3.3628 × 10^3^	3.4296 × 10^3^	3.3965 × 10^3^	2.3078 × 10^1^
	APO	3.3861 × 10^3^	3.5988 × 10^3^	3.4542 × 10^3^	5.0288 × 10^1^
$F_{30}$ (3000)	mAPO	6.4348 × 10^3^	2.1911 × 10^4^	9.1225 × 10^3^	3.2126 × 10^3^
	APO	6.6109 × 10^3^	2.1843 × 10^4^	1.0480 × 10^4^	3.4303 × 10^3^

**Table 4:** Statistical results obtained from mAPO, APO, SRA, HSO, EHO and YDSE for chaotic Rössler system.

Optimizer	Best	Worst	Mean	SD
mAPO	2.9986 × 10^−32^	4.6379 × 10^−31^	9.1664 × 10^−32^	9.0496 × 10^−32^
APO	4.3766 × 10^−23^	1.6346 × 10^−20^	2.1701 × 10^−21^	4.0528 × 10^−21^
SRA	1.0447 × 10^−12^	7.2703 × 10^−9^	8.2746 × 10^−10^	1.8884 × 10^−9^
HSO	4.9730 × 10^−6^	1.3154 × 10^−3^	2.3649 × 10^−4^	3.3537 × 10^−4^
EHO	9.3619 × 10^−23^	3.4515 × 10^−5^	4.0782 × 10^−6^	8.7899 × 10^−6^
YDSE	1.8141 × 10^−15^	2.6021 × 10^−12^	3.4006 × 10^−13^	6.1355 × 10^−13^

**Table 5:** Best obtained parameters obtained via mAPO, APO, SRA, HSO, EHO and YDSE for chaotic Rössler system.

Optimizer	Best Result			
	J	σ	ρ	β
mAPO	2.9986 × 10^−32^	0.200000000000	0.200000000000	5.700000000000
APO	4.3766 × 10^−23^	0.199999999997	0.200000000042	5.700000000179
SRA	1.0447 × 10^−12^	0.199999556380	0.200006233904	5.700038366982
HSO	4.9730 × 10^−6^	0.199447633752	0.196185393223	5.725533109820
EHO	9.3619 × 10^−23^	0.199999999996	0.200000000055	5.700000000367
YDSE	1.8141 × 10^−15^	0.200000013380	0.199999881661	5.700000061680

**Table 6:** Error rates achieved via mAPO, APO, SRA, HSO, EHO and YDSE for chaotic Rössler system.

Optimizer	Error Rate (%)		
	σ	ρ	β
mAPO	5.5511 × 10^−14^	1.3878 × 10^−14^	0
APO	1.5000 × 10^−9^	2.1000 × 10^−8^	3.1403 × 10^−9^
SRA	2.2181 × 10^−4^	3.1170 × 10^−3^	6.7310 × 10^−4^
HSO	2.7618 × 10^−1^	1.9073	4.4795 × 10^−1^
EHO	2.0000 × 10^−9^	2.7500 × 10^−8^	6.4386 × 10^−9^
YDSE	6.6900 × 10^−6^	5.9170 × 10^−5^	1.0821 × 10^−6^

**Table 7:** Comparison with the reported approaches for chaotic Rössler system in terms of best obtained parameters.

Optimizer	Best Result			
	J	σ	ρ	β
mAPO	2.9986 × 10^−32^	0.2000	0.2000	5.7000
FPA	4.1654 × 10^−7^	0.2000	0.2000	5.7000
IFFO	2.0942 × 10^−7^	0.2000	0.2000	5.7000
QPSO	2.1341 × 10^−11^	0.2000	0.2000	5.7000

**Table 8:** Comparative actual and estimated values of σ, ρ and β obtained via mAPO for different parts located in Figure 12, Figure 13 and Figure 14.

mAPO Optimizer Results		σ	ρ	β
Part (i)	Actual	0.2	0.2	5.7
	Estimated	0.2	0.2	5.7
Part (ii)	Actual	0.3	0.4	3
	Estimated	0.3	0.4	3
Part (iii)	Actual	0.5	0.3	8
	Estimated	0.5	0.3	8

**Table 9:** Statistical results obtained from mAPO, APO, SRA, HSO, EHO and YDSE for PMSM system.

Optimizer	Best	Worst	Mean	SD
mAPO	0	0	0	0
APO	1.4030 × 10^−26^	7.8780 × 10^−23^	4.9304 × 10^−24^	1.7485 × 10^−23^
SRA	1.9927 × 10^−16^	5.0394 × 10^−13^	6.3643 × 10^−14^	1.1125 × 10^−13^
HSO	4.9905 × 10^−10^	1.9264 × 10^−6^	1.9543 × 10^−7^	4.3346 × 10^−7^
EHO	1.8692 × 10^−11^	4.0328 × 10^−3^	4.1232 × 10^−4^	9.7369 × 10^−4^
YDSE	5.7472 × 10^−19^	1.9940 × 10^−15^	2.3200 × 10^−16^	4.6163 × 10^−16^

**Table 10:** Best obtained parameters obtained via mAPO, APO, SRA, HSO, EHO and YDSE for PMSM system.

Optimizer	Best Result		
	J	σ	ρ
mAPO	0	20.000000000000	5.460000000000
APO	1.4030 × 10^−26^	19.999999999999	5.460000000000
SRA	1.9927 × 10^−16^	19.999999690456	5.459999768468
HSO	4.9905 × 10^−10^	20.000454201311	5.460238731427
EHO	1.8692 × 10^−11^	20.000120412836	5.460077870351
YDSE	5.7472 × 10^−19^	20.000000017084	5.460000009443

**Table 11:** Error rates achieved via mAPO, APO, SRA, HSO, EHO and YDSE for PMSM system.

Optimizer	Error Rate (%)	
	σ	ρ
mAPO	0	0
APO	5.7554 × 10^−12^	6.3116 × 10^−12^
SRA	1.5477 × 10^−6^	4.2405 × 10^−6^
HSO	2.2710 × 10^−3^	4.3724 × 10^−3^
EHO	6.0206 × 10^−4^	1.4262 × 10^−3^
YDSE	8.5420 × 10^−8^	1.7295 × 10^−7^

**Table 12:** Comparison with the reported approaches for PMSM system in terms of best obtained parameters.

Optimizer	Best Result		
	J	σ	ρ
mAPO	0	20.0000	5.4600
ILCOA	9.8380 × 10^−30^	20.0000	5.4600
DE/ABC	2.9391 × 10^−27^	20.0000	5.4600
GA	0.0180	19.9593	5.4749

**Table 13:** Comparative actual and estimated values of σ and ρ obtained via mAPO for different parts located in Figure 18 and Figure 19.

mAPO Optimizer Results		σ	ρ
Part (i)	Actual	20	5.46
	Estimated	20	5.46
Part (ii)	Actual	25	7
	Estimated	25	7
Part (iii)	Actual	15	3
	Estimated	15	3

## Data Availability

All related data are presented within the manuscript.
